# Points to consider for incorporating climate impacts into health economic evaluation

**DOI:** 10.1007/s10198-025-01859-3

**Published:** 2026-01-07

**Authors:** Jule Oldenburg, Oliver Lange, Mattis Keil, Scott McAlister, Rachael Morton, Don Husereau, Wolf Rogowski

**Affiliations:** 1 Department of Health Care Management, Institute of Public Health and Nursing Research, Health Sciences, University of Bremen, 28359 Bremen, Germany; 2 Leibniz ScienceCampus Digital Public Health Bremen, 28359 Bremen, Germany; 3 Joint Research Cluster “Healthy City Bremen” of the University of Bremen, Bremen University of Applied Sciences and Apollon University of Applied Sciences Bremen, Bremen, Germany; 4 Faculty of Medicine, Dentistry and Health Sciences, The University of Melbourne, Melbourne, Australia; 5 Faculty of Medicine and Health, The University of Sydney, Sydney, Australia; 6 School of Epidemiology and Public Health, University of Ottawa, Ottawa, ON Canada; 7 Institute of Health Economics, Edmonton, AB Canada

**Keywords:** Health economic evaluation, CHEERS, Climate footprint, Climate impacts, External costs, Life cycle assessment, I19 Other, I18 Government Policy, Regulation, Public Health, I11 Analysis of Health Care Markets, I13 Health Insurance, Public and Private, D62 Welfare Economics – Externalities, D61Welfare Economics – Allocative Efficiency, Cost-Benefit Analysis, Q51 Valuation of Environmental Effects, Q54 Climate, Natural Disasters and Their Management, Global Warming, Q56 Environment and Development, Environment and Trade, Sustainability, Environmental Accounts and Accounting, Environmental Equity, Population Growth

## Abstract

**Objectives:**

Methodological approaches for incorporating the external effects resulting from climate impacts into health economic evaluation (HEE) are a vivid field of research. Combining established standards for reporting HEE and climate footprints (CF), our aim is to develop a structured list of points to consider for reporting full HEE that combines the two methodologies, referred to as climate-extended HEE

**Methods:**

We mapped a transparency catalogue with methodological items for estimating CF to the reporting items described in the Consolidated Health Economic Evaluation Reporting Standards (CHEERS). We identified synergies and developed a proposal of methodological points to report for climate-extended HEE, structured by the CHEERS items. The proposal was validated using three published climate-extended HEEs and a hypothetical case study..

**Results:**

We proposed extensions to 18 reporting items of CHEERS, for example, adding more detail to the measurement and valuation of resources and costs to facilitate a process- or cost-based estimation of CF. Using three identified publications and a hypothetical case study, examples on how all items could be addressed are provided, including a presentation of climate-extended versions of the standard summary measures of HEE.

**Conclusions:**

The proposed catalogue can be used for reporting and reviewing climate-extended HEEs. Further work is necessary to include planetary boundaries beyond climate change. Future steps could be, first, to develop a reporting standard within a formal Delphi process of all relevant stakeholders. Second, the catalogue can be used to develop standards of analytic choices for specific decision makers or problems.

**Supplementary Information:**

The online version contains supplementary material available at 10.1007/s10198-025-01859-3.

## Introduction

The methods of health economic evaluation (HEE), i.e., the comparative analysis of health interventions in terms of costs and consequences [see 1], are constantly developing. Accordingly, also HEE reporting standards are expanding or newly evolving to raise awareness about blind spots of previous studies and to guide new ones to account for the methodological developments in a transparent manner. For example, in recent years, besides efficiency, also concerns about prioritizing the more severely ill or about reducing social inequality in health have received increased attention in HEE [see e.g. 2]. As a consequence, the Consolidated Health Economic Evaluation Reporting Standard (CHEERS) [3, 4] was extended to include an item (CHEERS item #19) that requires characterizing distributional effects [5]. Or, new methods like expert elicitation for generating parameter distributions or value of information analysis for estimating the potential benefit of additional evidence were introduced for HEE, and consequently, reporting standards for using expert judgment [6] or estimating value of information [7] were developed.

A methodological topic that has received substantial interest recently is how to incorporate negative environmental external effects into HEE. One of them is global warming: The healthcare sector emits 4.4% of global greenhouse gases (GHG) and is thus one major contributor to climate change [8], with more than two-thirds of these emissions originating from the supply chain (so-called scope three emissions [9, 10]). Governments have committed to reducing GHG emissions from their health systems, exemplified by initiatives like the Alliance for Transformative Action and Health, aiming for low or net-zero emissions in the health sector [11, 12]. From April 2028, National Health Service (NHS) suppliers will need to report the GHG emissions of their products to support the reduction of scope 3 emissions in the NHS [13]. Also, incorporating the external costs of GHG emissions into budget decision-making is becoming mandatory for many public bodies (e.g., in Germany, [14, 15]). A survey of health technology assessment agencies by Bobini and Cicchetti [16] revealed that most of the agencies have either made attempts to or are now working on integrating environmental effects into their evaluation processes.

There are a number of recent conceptual studies that provide insights into how environmental impacts can be integrated in HEE as outcomes or as costs theoretically [17–24] or practically [25–34]. As the global impact of climate change is directly affecting human health, it could also be argued that it is critical no longer consider the impact of GHG emissions as an externality. Various recent systematic and scoping reviews have explored methods to incorporate environmental impacts into HEE [35–39], with partly overlapping articles. In their systematic review, Williams, Bell [36] report ten different approaches to incorporate environmental impacts into HEE or health technology assessments. These range from integrated approaches such as incorporating environmental impacts as costs [19, 25, 28, 29, 40–44] or outcomes [21, 28, 40], to separate approaches that use the Incremental Carbon Footprint Effectiveness Ratio (ICFER) [32] or Incremental Carbon Footprint Cost Ratio [32]. Also, they can be integrated using multi-criteria decision analyses [19, 40, 41, 45], evaluated separately alongside HEE [19, 40, 41, 45], reported without further evaluation (information conduit) [40] and/or be included in deliberative decision-making processes [24, 40–42]. Different health economic study types were identified that provide examples of how external costs of climate change can be incorporated: Budget impact analyses [46, 47], cost of illness analyses [28, 29], and health economic evaluations [25, 32]. Among various aspects that require further work to facilitate systematic incorporation of external environmental costs into HEE, one is methodological standardization [37], Guirado-Fuentes, Abt-Sacks [37], Pinho-Gomes, Yoo [38], also to prevent potential greenwashing [37]. One possible solution, as Carrandi, Nguyen [39] emphasize, is the development of guidelines for reporting environmental impacts in HEEs.

**Fig. 1:**
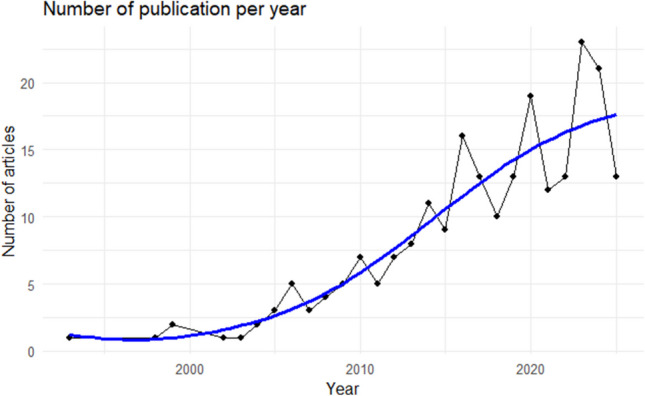
Number of publications addressing climate-extended HEE, either conceptually or through applied analysis

Figure 1 provides an overview of studies per year, analyzed with the R package Bibliometrix [48] (further details on search and analysis are provided in Supplementary file). The declining trend may indicate that after a period of increasing numbers of new methodological ideas, now, it appears timely to consolidate these ideas and start developing standards for conducting and reporting HEE that incorporate external environmental impacts.

Despite the differences of the studies, there is one shared methodological point of orientation for integrating environmental impacts into HEE: the use of life cycle assessment (LCA). LCA is a widely used, broad methodological approach for measuring and valuing environmental impacts during a product’s creation, use, and disposal [49]. The way LCAs are undertaken and reported have been standardized by the International Organization for Standardization in ISO 14040 and 14044 [50, 51]. While LCAs quantify a range of environmental impacts, in the following, we focus solely on climate impact, since it is particularly relevant for decision-making with regard to climate targets, and it is mainly the focus of existing studies dedicated to the integration of environmental impacts into HEE [35, 36].

A metric for assessing climate impact is the climate footprint (CF), measured in kg CO_2_ equivalent (CO_2_eq). CF measures the effect of CO_2_ and other GHGs, such as methane, nitrous oxide, on global warming. While this metric is also commonly called “carbon footprint”, it can be argued that the latter term should refer to CO_2_ only [52].

ISO 14040 and 14044 underpins three CF standards. ISO 14067 [53] and the British Standards Publicly Available Specification (PAS) 2050 [54] focus on products and services, while the Greenhouse Gas Protocol’s Corporate, and Products, standards provides guidance for both organizations and products [55]. These standards differ in methodological considerations [see e.g. 56 as well as in their requirements for reporting methodological choices. However, they contain a shared methodological items that were recently synthesized in a checklist for assessing the transparency of CF studies to facilitate systematic reporting and data extraction for systematic reviews [57].

Assessing the whole life-cycle, the material a product or service consists of needs to be connected to material and emission flows before and after its production – ideally within all the phases of raw material extraction, production, transport, use and disposal (“cradle to grave”). One of the various methodological choices in an LCA is the data collection and source for those upstream production processes. It requires balancing precision and feasibility. Process-based data includes product-specific data on material flows over the life cycle which can either be collected by direct measurement or can be taken from background data bases like Ecoinvent [58]. Usually, process-based data refers to an output based on physical units (e.g., an average medical consultation [59] or one kg of stainless steel) and the flows of physical resources it involves. Even if this is most precise, process-based data is not available for all products.

An alternative is using emission factors from environmentally-extended input–output (EEIO) tables [60]. Input–output tables track the value of goods across industries of all major economies, EEIOs add emissions to these values. They thus provide information on aggregated industry sectors (such as the manufacture of metal products or the manufacture of chemicals). Given that they are constructed in a top-down approach so that the sum of sectors equates to the economy in total, the tables can provide information on emissions for all production and service sectors. As EEIO tables measure sector outputs in monetary units, the information is provided as cost-based emission factors (e.g., 300 g CO_2_eq per € output from the pharmaceutical sector taken from EXIOBASE according to Stadler [61]).

However, there can be substantial differences between process-based and EEIO CF estimates [62]. The limitations of EEIO are of particular importance for healthcare, e.g. because of administrative prices or because price differences between patented and generic medical products are hardly linked to differences of environmental impacts. Additionally, results differ depending on whether all environmental impacts are estimated that can be attributed to a good, or whether the estimation focuses on impacts that arise as a consequence of the decision to use a product (attributional vs. consequential LCA [see e.g. 63]). For example, MRI scanners require substantial amounts of electricity to keep their magnets cool but the additional electricity required for an additional CT image is comparatively low. McAlister, Barratt [64] illustrate that this results in a difference of 77 kg CO_2_eq (EEIO/attributional) compared with 1.1 kg CO_2_eq (consequential).

To address the limitations of EEIO-LCA at a product level, EEIO is often used as a complement to process-based LCAs [65, 66]. This approach, known as hybrid LCA, remains a topic of debate. In particular, there is an ongoing discussion about whether combining process-based LCA and EEIO methods adds complexity without significant precision gains. However, due to the lack of data on the CF of health services, both process- and cost-based approaches have been used as starting points for improving the evidence on the climate impacts of healthcare and public health [e.g. 34, 67].

There are multiple connecting points between HEE and LCA: For example, properly estimating the CF of a new medical intervention involves estimating the intervention’s effects and associated resource use and savings (e.g., the impact of additional devices and their use or of carbon-intensive surgery avoided by a digital intervention to prevent cardiac diseases [68–71]). However, to date, neither standards for reporting nor for conducting HEE are yet suited to address the various methodological issues involved with properly assessing climate impacts. To facilitate the incorporation of CF into HEE, there is thus a need to map relevant items of the two methodologies and develop a consolidated catalogue that can be used both for reporting and assessing transparency, as well as a basis for discussing and determining analytic choices to be made for each of the items.

Our aim is to provide input to the development of a reporting standard for HEE that aims at incorporating climate impacts (which we refer to as climate-extended HEE). Consequently, we explore how the existing reporting framework can be leveraged and where its reporting scope could be extended to enhance transparency in the reporting of climate-extended HEE. To demonstrate the feasibility of this approach, we apply the identified points to consider to a published full HEE that reports both costs and consequences, including climate impacts, as well as to a hypothetical case study.

## Methods

Based on the related literature, we combined methodological reporting items for HEE and estimating CF. As a reference reporting standard for estimating CF, we chose the recently published transparency checklist for CF by Lange et al. [72] (in the following “CF-checklist”) because it integrates the reporting requirements of the three most widely used CF standards; ISO 14040 and 14044, the GHG Protocol and PAS 2050 [54]. Like CHEERS, it may, therefore, serve as a methodological reference for points to consider in estimating CF and is thus not tied to one specific set of methodological choices. Second, we assessed which CF-checklist items are already contained in CHEERS (even if the wording may differ). Third, for the remaining items in the CF-checklist, we searched for thematic proximity with CHEERS items to determine where these methodological items can most suitably be added to established HEE reporting items. Fourth, we investigated the DIN EN ISO 14067 to explore potential items for integration in addition to the existing information. Finally, for CHEERS items not yet complemented by CF analysis, we explored the literature to determine whether there are remaining links between CF calculation and HEE that are worth integrating into these revised methodological items of CHEERS.

The process started with a first mapping by the author JO and was discussed in an iterative process by the authors WR, OL, MK, and JO. Then, JO and OL proposed a draft of points to consider for reporting climate-extended HEE. These concepts were socialized with RLM, a health economist working closely with HTA agencies and Government in the Southern Hemisphere and SMA, a senior LCA expert focusing on healthcare. All of the authors discussed the results until a consensus was reached. To validate and refine the proposed points to consider, a set of studies identified in recent reviews on climate-extended HEE [35–39] was used as reference examples.

In addition, a new, hypothetical case study was developed which assesses climate-extended cost-effectiveness of four interventions for preventing diabetes in individuals with obesity: A – health promotion with a focus on plant-based diet, B – intensive nutritional counselling for planetary health diet, C – CO_2_ intensive formula diet, D – psychotherapy aimed at behavioral change towards a low carb diet. Using hypothetical values for health effects, costs, and climate impacts, the case study served for testing the feasibility of fully transparent reporting and for illustrating how different health economic summary measures can be established in a climate-extended manner. Details for the case study are provided in the Supplementary file.

## Results

### Results of matching

**Fig. 2:**
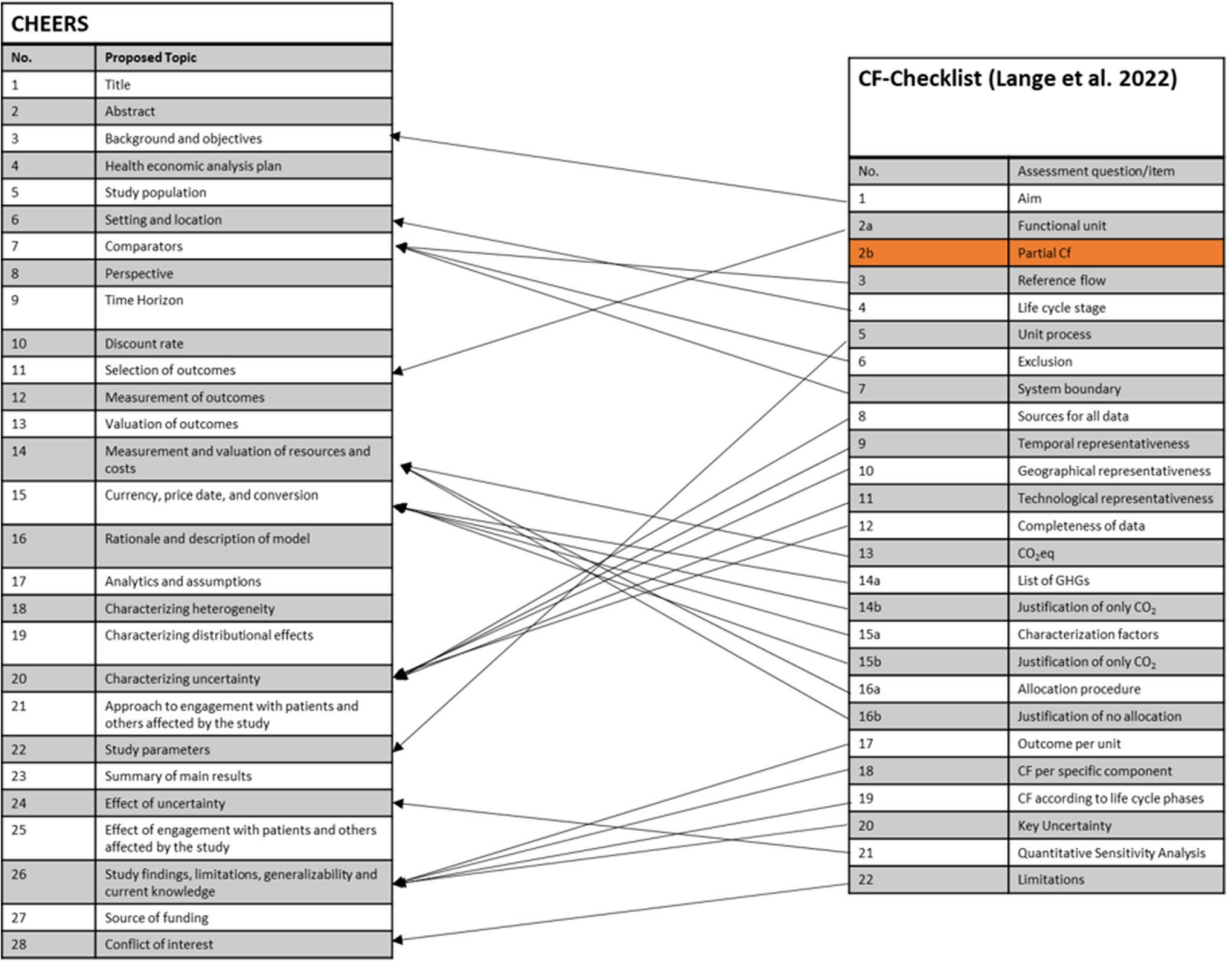
Matching of the CF-checklist into CHEERS

Figure 2 illustrates the matching process of the items from the CF-checklist of Lange et al. [72] to CHEERS. We incorporated all items of the CF-checklist into 11 items of CHEERS. Item 2b of the CF-lists was excluded because partial CFs only assess the CF of some product or service without reference to its purpose. In contrast, CHEERS requires reporting not only resources/costs but also outcomes associated with the interventions, which corresponds with full CF.

### Results of the study identification

Three published full climate-extended HEE could be extracted from the reviews: de Preux and Rizmie [25] who assessed in-centre versus home haemodialysis (reviews: [35, 38]), Kindred, Shabrina [32] who used obesity interventions as case studies (review: [36]), and Smith, Tennison [73] who assessed the carbon footprint and climate-extended cost-effectiveness of different smoking cessation services (review: [37]).

### Points to consider when incorporating climate impacts

Below are brief explanations of the identified methodological points to report when climate impacts are incorporated into HEE. For CHEERS items #4, #21, #25, #27, and #28, no relevant methodological amendments were identified that generally require reporting for climate-extended HEE, and thus, they are not discussed in the following paragraphs.

Furthermore, for items #5 and #16-#19, it may occur that specific information ought to be provided, but it is not generally the case. For example, even if LCA can be seen as a modelling method on its own, adding information under item #16 is only necessary to the extent that specific modelling of CF was conducted in excess of the LCA model, which was the source of CF of single items as reported under item # 14. These items were excluded in the following as well.

### Title, abstract, and introduction of the evaluation

CHEERS requires that the title indicate the study as an economic evaluation and delineate the interventions under comparison (item #1). HEE abstracts should offer a structured summary highlighting context, key methods, results, and alternative analyses (item #2) [4]. Also, it is desirable for authors that climate-extended HEEs are identifiable through a database search of the title and abstract. The studies in this review used the terms “carbon footprint” [32, 73] or “environmental sustainability” in their titles; our own case study uses the expression “climate-extended cost-utility analysis”. All studies could be identified with a title and abstract search that includes “cost-effectiveness” and a term representing the relevant environmental impact. Titles and abstracts of the three published studies only provided limited information on the methods of the climate-extended economic evaluation. However, this may be due to the fact that the studies either were conceptual [25, 32] or that cost-effectiveness was taken from studies published separately [73].

Methodological decisions and assumptions of HEE have to be made depending on the research question and decision context (item #3) [4]. This corresponds with the importance of a goal and scope statement in LCA, which states the target audience, reason for carrying out the CF study, application, and whether or not the study involves a comparison to be published [74]. As an example of methodological consequences of the goal and scope statement, climate-extended HEE typically includes comparisons because this is an element of the standard definition of HEE [75, p. 4]. This involves the need to ensure using equivalent methodological considerations of CF estimation for all decision alternatives [76]. All three published climate-extended HEE state the UK’s NHS as context for the analysis and its Sustainable Development Unit (forerunner of today’s Greener NHS programme^1^) or its National Institute for Health and Care Excellence^2^ as the relevant decision body.

### Evaluation methods

#### Setting and comparators

HEE should offer important contextual information that may affect results (item #6) [4]. Considering climate impacts makes additional contextual information relevant, beyond factors like “ambulatory care”, “funded by the statutory health insurance”, or “in Germany”. Climate impacts extend beyond the health system over all five phases of a product’s life cycle (raw material extraction, manufacturing, distribution, use, disposal/recycling) as demonstrated by Sanchez, Eckelman [34], and McGain, Story [77]. Since these phases can take place in different regions, variations in electricity mix, mobility behavior, supply chains or trade relations may need consideration. Furthermore, climate impacts may vary between rural and urban settings, impacting, e.g., travel costs of fuel consumption for physicians. Hence, contextual information for all life cycle phases may have to be specified.

CHEERS item #7 requires reporting the interventions or strategies being compared [4]. Similarly, in CF analyses, describing the objects of investigation is also essential. Compared to standard information in HEE, CF analyses require additional information, such as the product system, the system boundary, and the reference flows. A product system is the totality of connected processes (mechanisms that convert inputs and outputs) and flows (material or energy that enter or leave the system under study) [53]. System boundaries are established on the basis of specific criteria to determine which processes to include into the life cycle model [53]. These criteria should be clearly described [78]. While some LCA modelers use standard cut-off criteria (such that processes that deliver impacts contributing less than 5% of the end product’s total impact), rather recommend excluding processes based on case-by-case justification. For example, in the case study (see Supplementary file ), GHG emissions due to the transport of the formula diet from the store to the user’s home were excluded because they are, first likely to be negligible, and, second highly uncertain. The starting point of modeling processes is the reference flow, which is the full list of resources (products, electricity, other inputs and outputs) required to fulfill the function of the system, i.e. to achieve the aim of the intervention [53]. “Reference flow” and “description of comparators” are thus very similar concepts, as both include a detailed specification of the intervention components.

#### Perspective & time horizon

Following CHEERS item #8 (Perspective), assessing “costs” always involves stating whose costs were incorporated. However, climate costs can be added to costs from any perspective and a climate-extended HEE can be conducted from a more narrow than societal (e.g., healthcare payer) perspective. To further differentiate, the perspectives of internal and external costs could be distinguished. Different perspectives of external costs could become relevant, for example, if local environmental impacts like emissions of soot particles of products manufactured in China and used in the EU were included. However, even if soot particles also have an impact on global warming, the GHGs which are typically considered in CF estimations primarily induce global impacts. Therefore, the perspective of external costs of climate change is typically global and societal without a need for specific reporting in different climate-extended HEE. Accordingly, it was reported in none of the published climate-extended HEE or just as limitation [cf. 32].

CHEERS item #9 requires stating the time horizon and justifying its appropriateness [4]. Determining global warming potential requires a methodological decision, which is similar to deciding about the time horizon in HEE. GHG emissions may persist over different durations, and their impact can vary over time due to factors like absorption or decomposition. Thus, two time horizons may therefore be necessary: one for intervention costs and effects, and another for the calculation of global warming potential. The choice of time horizon is made in conjunction with the choice of characterization factors (see item #15). Even though this topic is still discussed in the literature [79, 80], often, global warming potential over 100 years (“GWP100”) is selected. This may also be reasonable for HEE because the values are readily available and they are similar to the convention in HEE of using a lifetime horizon to account for long-term health gains. None of the published studies provided information on the GWP time horizon.

#### Discount rate

Item #10 mandates reporting the discount rate. Similar to cost data, emissions can be discounted to adjust for temporal preferences. The same is the case for both environmental costs and environmental impacts on health outcomes. Furthermore, the time of emission can be important as the effect of the GHGs is partly dependent on the GHG concentration in the atmosphere. If the emissions are connected with their climate costs (e.g., SC-CO_2_), it appears most consistent to apply the discount rate also in their calculation.

There is an ongoing debate on how to integrate the temporal dimension in LCA [81]. Some suggest avoiding discounting in single-product footprints for informational purposes, while others argue that discounting enhances comparability between products [82]. However, as noted in the review of Lueddeckens, Saling [81], no clear consensus has been reached. Discount rates and time preferences can be included in LCA methodology through the differentiation between static and dynamic models. Static models aggregate all emissions at a single point in time which implies assuming a discount rate of 0%, while dynamic models consider the time of emissions and differences in time between production steps, usage, and disposal in their inventory (item #14) and their characterization (item #15) [83]. Therefore, in dynamic models, a discount rate could be applied. However, typically, most LCAs and the databases they rely on use static models. Typically, a 0% discount rate is applied to avoid disadvantaging future generations [84]. The only published climate-extended HEE that reported discounting of its CF estimates [32] followed this convention.

#### Selection, measurement, and valuation of outcomes

Items #11, #12, and #13 require stating the types of outcomes and how they were measured and valued (if applicable) [4]. Selection of outcomes not only determines the type of HEE (e.g., if QALYs or DALYs were selected as outcomes, the HEE is termed cost-utility analysis [1]) but also influences an important methodological choice of any LCA: the definition of the functional unit. The functional unit represents the quantified performance of a product system (see also item #7). This is necessary to facilitate consistent comparisons between interventions [34, 53]. Given that the outcomes of HEE are also the points of reference for the climate impacts, they can be seen as health-specific types of functional units. Therefore, we use the concepts “definition of functional unit” and “selection of health outcomes” interchangeably (see also item #7).

Generally, accounting for climate impacts as outcomes may appear straightforward, as climate change also involves health impacts. However, unlike HEE, where the focus is on QALYs gained, to our knowledge, all LCA models that report health endpoints of environmental damages use DALYs as outcomes (e.g., ReCiPE [85], UseTox [86, 87], ILCD [76], and EcoIndicator99 [86–88]). Even if these DALY estimates are subject to substantial uncertainty, they allow for offsetting an intervention’s environmental impacts from its DALYs averted. Also, human health damage factors based on four climate change scenarios estimated by the Intergovernmental Panel on Climate Change (IPCC) could be used to estimate the health impacts of greenhouse gas emissions in terms of DALYs. The amount of DALY/kg CO_2_ that was computed for each scenario is$2.0x{10}^{-7}$,$6.2x{10}^{-7}$,$2.1x{10}^{-7}$, $4.2x{10}^{-7}$. However, then, additionally, the chosen scenario needs to be reported.

Additionally, if outcomes are incorporated as benefits, disability weights need to be reported, at least if they deviate from the standard of using the Global Burden of Disease Reports [89]. However, less than 10% of HEE use DALYs averted as outcomes [90]. Therefore, the comparison is difficult, and it is likely to be more convenient to focus on the cost side when incorporating climate impacts. None of the published climate-extended HEEs used DALYs as a measure of climate impact.

#### Measurement and valuation of resources and costs

CHEERS item #14 requires explaining how cost estimates were established [4]. Initially, Drummond, Sculpher [1] separated resource identification, measurement, and valuation. To include climate impacts, it may be advisable, following Pugliesi, Marrauld [23], to follow this conventional path of costing and first provide a list of resources in physical units, and separately report their monetary values as starting point for measuring climate impacts and valuing them if they are accounted for as costs.

This is because CF for each resource can be determined either using physical or monetary units. According to the Greenhouse Gas Protocol, three different calculation approaches are available: product-specific process-based data; average, process-based data of similar products (databases, literature); and cost-based emission factors. Given the different measurement methods, outcomes for the same product are likely to differ, so reporting the measurement method is essential.

In the first and second approaches, emissions from LCA databases or from published LCAs are assigned to the resources in physical units. Alternatively, GHG emissions can be calculated from valued resources using cost-based emission factors from EEIO-LCA. This third approach may introduce higher uncertainty due to aggregation errors, though [60]. These errors are likely to be particularly high if reimbursement rates are the basis of climate costing because they may cover very different types of technology or human activities that may involve very different emissions.

Resources may serve many different departments, programs, or processes—for example, general hospital administration or power consumption – so that their costs need to be attributed to specific interventions using some mechanism of allocation [1] [p. 218ff.]. Similarly, to the allocation of overhead impacts to cost units, GHG emissions may also need to be allocated to different activities [53], and the allocation methods need to be reported.

Besides estimating health outcomes of CF, the harms of GHG emissions can be expressed by estimates of the social costs of carbon per CO_2_eq/year (SC-CO_2_), which “measures the monetarized value of the damages to society caused by an additional metric ton of CO_2_ emission” [91]. Although there are standards for determining environmental costs of organizations [92] and for evaluating monetarized environmental harms [93], there is no standardized value [19], also due to uncertainty [94–96]. Therefore, different values may be used (in Germany, for example, €237 SC-CO_2_ is recommended [97]). The climate costs of other GHGs, such as methane and nitrous oxide, can be calculated by multiplying by the SC-CO_2_ with the global warming potential of a GHG, i.e., its impact on global warming compared with CO_2_ [53]. For this calculation, the amount of the GHG and its global warming potential are needed, which can be obtained from Myhre, Shindell [80]. To ensure comparability and transparency, the chosen SC-CO_2_ value ought to be reported.

Any estimate of external emission costs needs to consider the extent to which these costs are indeed external, and not yet included in market prices. This is represented by the non-traded price of carbon, which excludes emissions subject to existing taxation, such as carbon taxes or emissions trading systems [25]. According to the Intergovernmental Panel on Climate Change, 20% of the GHG emissions were captured by a carbon tax or an emission trading scheme in 2020 [98]. This could be accounted for, e.g. estimating total cost of GHG emissions based on the amount of CO2eq multiplied by the SC-CO2 [53] and reducing this value by 20%. Alternatively, in cases where GHGs are not aggregated to CO_2_eq, a price can be assigned to each GHG, justified by the fact that GHGs other than CO_2_ are not currently subject to taxation. This could either be based on the product of SC-CO_2_ and the GWP of each GHG, or on specific social costs of other GHGs, like the social cost of methane or nitrous oxide. However, recommendations on the SC of other GHG are still evolving (see for example [99, 100]). Adjusting for the non-traded price of GHG only for CO_2_ overlooks the potential effects of the trading systems in regions like California, Korea, and Switzerland, which also cover other GHG [101]. This illustrates that it is essential to transparently report which GHGs are considered and valued and how trading system or tax coverage is accounted for.

All of the published studies only reported literature references to substantiate their estimates of the amounts of GHGs rather than methodological details. For valuation, de Preux and Rizmie [25] used a non-traded price of £52 per ton CO_2_eq in the United Kingdom to measure the healthcare sector externalities. Smith, Tennison [73] used an uncorrected social cost of £28/t C02e and Kindred, Shabrina [32] calculated summary measures without assigning DALY or currency values to the CF estimates.

#### Currency, price date, and conversion

CHEERS item #15 requires reporting the currency used, price date, and method of conversion [4]. Similar to different currencies, which can be used for monetary valuation and conversion, different metrics are available for determining the climate impact of GHGs. Rather than calculating each GHG’s impact on global warming individually, it is more practical to transform emissions into a common unit, CO_2_eq. The characterization factor of a GHG specifies its global warming potential compared with that of CO_2_ [53]. Characterization factors are based on a chosen time horizon (i.e., warming potential over the next 20, 100, or 1000 years, see item # 9, time horizon). The IPCC recommends including six types of GHGs: carbon dioxide, methane, nitrous oxide, hydrofluorocarbons, perfluorocarbons, and sulfur hexafluoride [102]. For characterization factors, established databases exist, such as the IPCC´s Emission Factor Database [103] or models such as the ReCiPe [104]. None of the published studies reported on this item.

#### Characterizing uncertainty and the effect of uncertainty

CHEERS item #20 requires describing the methods employed to characterize any sources of uncertainty within the analysis [4]. When considering climate impacts, new uncertainties arise that ought to be addressed. ISO Standard 14067 outlines requirements for assessing data quality, including time-related, geographical, and technology representativeness (i.e., whether CF estimates adequately represent the current state of technologies, local conditions like national energy mix, or the type of technologies actually used), completeness, consistency, and reproducibility [53].

Similar to HEE modeling standards, the Monte-Carlo simulation is one standard tool for quantifying the joint impact of parameter uncertainty on LCA results. If distributions are unknown for single parameters that are used in the CF model, an approach termed the Pedigree matrix is a frequently proposed tool to estimate the parameters’ standard deviation. It uses a qualitative rating of reliability, completeness, temporal representativeness, geographical representativeness, and technological representativeness. Based on whether the criteria are rated very good, good, fair, or poor, pre-specified uncertainty scaling factors are assigned, and standard deviation is estimated using a standard exponential formula [105]. Alternatively, data uncertainty can be described qualitatively [76]. However, many LCA studies do not conduct an uncertainty analysis, making it more challenging to include the results as parameter distributions in HEE and report them [106]. None of the published climate-extended HEE provided a comprehensive characterization of time-related, geographical, and temporal representativeness and conducted deterministic sensitivity analyses. Kindred, Shabrina [32] include a distribution of CF in their probabilistic analysis.

## Evaluation results

CHEERS item #22 requires reporting all study parameters (values, ranges, and references including uncertainty or distributional assumptions) [4]. In LCA, reporting all analytic inputs is typically done through the Life Cycle Inventory, which presents all inputs and outputs for a product system in an inventory. Given the connection between resources and emissions (see item #14), it appears feasible to include emission estimates and their parameter distributions in tables reporting cost estimates in standard HEE. Among the three published studies, only Kindred, Shabrina [32] include one distribution of CF by assuming a 5% standard error for the NHS carbon intensity of 0.156 kgCO2e/£.

Item #23 asks for reporting mean values for the categories of outcomes of interest and whether they are summarized in the most suitable overall measure [4]. In this context, both effects and costs could be reported with and without climate impacts. If overall measures such as incremental cost-effectiveness ratios (ICER) [1] are appropriate for a given analysis, there are different options to include climate impacts here. For example, as a non-monetary measure, the incremental carbon (or climate) footprint effectiveness ratio (ICFER) can be calculated, which assesses additional health outcomes gained per additional ton of CO_2_eq emitted. Also, the incremental carbon (or climate) footprint cost ratio (ICFCR) can be presented, i.e., the cumulative CF per cumulative currency unit spent [32]. Additionally, corresponding with the sections on climate costs above, ICERs could be reported with a climate cost extension (ICER-CCE) if climate costs are added to the costs from conventional HEE. Likewise, net monetary benefits (NMB) based on incremental costs, effects, and a threshold value [1] can be climate-cost-extended (NMB-CCE) by adding climate costs to the conventional costs. Also, net benefits from cost–benefit analysis can be climate-extended in the same way. If damage from climate change is included on the outcome side, then the ICER-climate-effect-extended (ICER-CEE) or the NMB-climate-effect-extended (NMB-CEE) could be reported, in which outcomes associated with climate footprint are subtracted from the interventions’ benefits.

Table 1 illustrates ICER, NMB, ICFER, ICER-CCE, NMB-CCE, ICER-CEE, and NMB-CEE for the hypothetical case study of diabetes prevention in individuals with obesity. The interventions include: S) standard care of no intervention; A) health promotion with a focus on a plant-based diet; B) intensive nutritional counselling for a planetary health diet; C) CO_2_ intensive formula diet; and D) psychotherapy aimed at behavioral change towards healthier diet without reducing meat and dairy products. The GHG values are derived from Clay, Charlton [107].

**Table 1:** Comparison of ICER, NMB, ICFER, ICER-CCE, NMB-CCE, ICER-CEE, NMB-CEE

ΔDALYs	ΔCosts	ICER	NMB	ΔtCO2eq	ICFER	Costs-CCE	ICER-CCE	NMB-CCE	DALYS-CO2	DALY CEE	ICER-CEE	NMB-CEE
0	0	N.A	N. A	0	N.A	0	N. A	N. A	0	N. A	N. A	N. A
1.5	25,000	extendedly dominated	95,000	−4	dominant	24,242		95,758	0.003712	1.504	extendedly dominated	95,297
2.7	37,000	dominated	179,000	−40	dominant	29,416	10,895	186,584	0.03712	2.737	dominated	181,970
3	30,000	10,000	210,000	20	dominated	33,792	14,587	206,208	0.01856	2.981	10,062	208,515
3.1	50,000	200,000	198,000	2	0.65	50,379	165,872	197,621	0.001856	3.098	171,374	197,852

Although there are various estimates for both, outcomes and costs, it should be clearly reported which values are chosen, ideally in accordance with guidelines like the ISO 14007/14008 [92, 93]. For DALYs, the case study used the ReCiPe impact assessment methodology, which is one of the most widely used impact assessment methods [108]. However, certain environmental effects of climate change, such as droughts, are not considered in the DALYs calculation [85], emphasizing the need for a standardized value and transparent reporting also regarding the choice of impact assessment model. Although various cost estimates exist, which tend to increase over time and depending on the extent of climate damage included [109], our case study followed the recommendation by Fraas, Graham [110] to use a country specific value. Thus, for Germany, a hypothetical non-traded price of carbon of 189,6€, based on estimates of Matthey and Bünger [111] and Shukla, Skea [112], was chosen. For illustration purposes, a mean value of €80,000/DALY was applied as cost-effectiveness threshold for Germany, as recommended by the UN, using two times GDP per capita as reference [113]. Of course, it needs to be acknowledged that such thresholds have to be handled with care [114, 115].

Moreover, a critical challenge in integrating climate impacts into HEE is the potential trade-offs, that may arise between an intervention´s effectiveness and its environmental impact. Desterbecq and Tubeuf [35] argue that applying the equity-efficiency trade-offs used in HTA provides a practical approach to address these challenges.

**Fig. 3:**
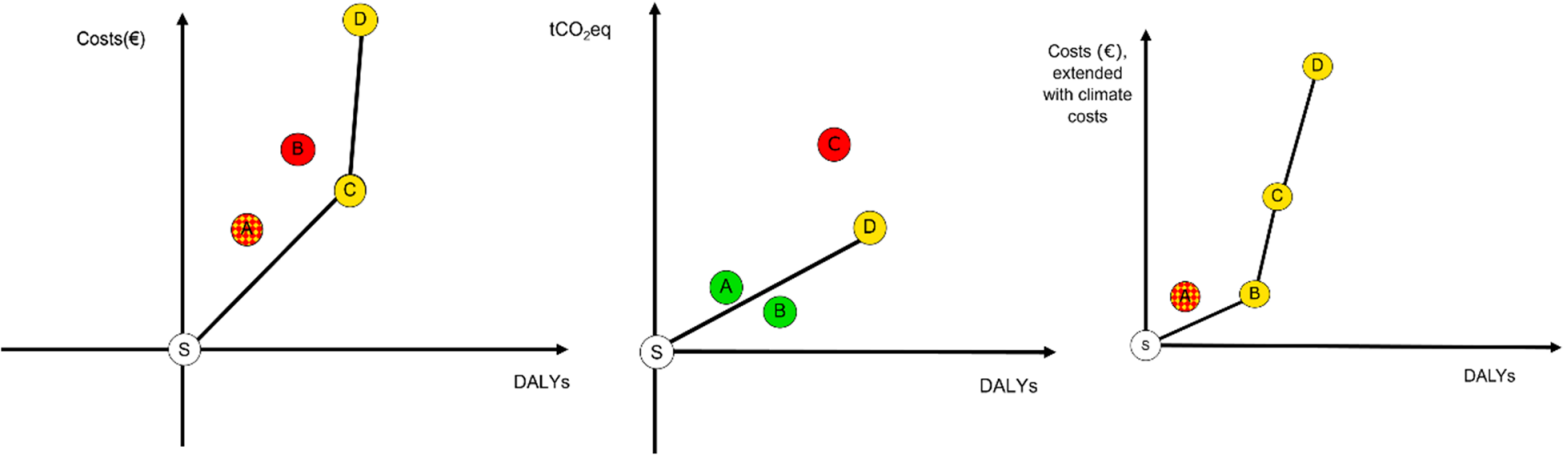
ICER, ICFER, and ICER-CCE for four hypothetical case studies

The case study calculation demonstrates the multitude of available summary measures for including climate impacts and how the inclusion may change the results of HEE: taking climate impacts into account, Intervention B appears to be the most cost-effective, which would not have been the case without this inclusion. It also illustrates differences depending on whether climate impacts are included in the costs or the effects side. On the effect side, the impact on the case study is smaller, which is consistent with the fact that health damage in terms of DALYs are only one part of the social costs of carbon.^3^ Figure 3 presents the differences graphically.

All published climate-extended HEE reported the CF estimates of the different interventions, only Smith, Tennison [73] report the contribution of different sub-processes to total CF (e.g., that patient travel was the largest source of emissions with a share of 60% for group counselling). As summary measures, de Preux and Rizmie [25] and Smith, Tennison [73] report ICER-CEE values. Kindred, Shabrina [32] report ICFER values as well as (negative) ICFCR values (i.e., CF savings per £ spent).

Item #24 requires describing how uncertainty regarding analytic judgments, inputs, or projections influences findings, along with reporting the impact of the choice of discount rate and time horizon. Item #20 requires characterizing sources of uncertainty also for CF analyses; here, their impact on the results ought to be reported. Also, it includes the impact of methodological uncertainty like whether the impact of GHG emissions is included on the costs or effect side. None of the three published studies reports the impact of all LCA-relevant analytic choices. Smith, Tennison [73] report the impact of staff commute on the overall CF; Kindred, Shabrina [32] report different scenarios and probabilistic results, including the probabilistic estimate of CF.

## Reporting key findings, limitations, generalizability, and current knowledge

Item #26 requires reporting key findings, limitations, generalizability, and current knowledge. The limitations to be reported for the climate footprint are defined by the ISO 14067 [53]. The two most important limitations include focusing solely on climate change as the single impact category and methodological constraints such as choice of system boundary and functional unit, data quality and sources, allocation procedures, and assumptions regarding issues like transport, user behavior, or end-of-life scenarios [53]. All three published studies discuss limitations also regarding their CF analysis; however, none discuss the items to be addressed according to the ISO Norm 14,067 [53].

## Discussion

### Interpretation of the results

There are various links between methods of HEE and CF, which generally provide a basis for transparently including GHG emissions as impacts into climate-extended cost-effectiveness analysis. This incorporation of climate impacts can help decision makers who aim at prioritizing climate-friendly interventions to do so in an economically consistent manner, potentially reducing emissions in the public health sector.

Similar to measuring health outcomes, the estimates of environmental outcomes depend on the methods of how calculate CF. Therefore, transparent documentation of methodological choices is essential. In some cases, the complexity of the intervention or its expected CF may require conducting full LCAs and reporting it in one or more separate publications. In other cases, adding CF to HEE in a pragmatic manner based on a combination of pre-established standard carbon costs and emission factors with data from existing LCAs and LCA database entries may appear the most reasonable alternative to ignoring climate impacts. We aimed to provide a reporting framework that can be used for transparently integrating CF into HEE in both cases, as it allows very different methodological choices and levels of sophistication.

### Limitations

This research only includes GHG and does not encompass other environmental effects that are measurable by LCA like impacts on the other of the nine boundaries proposed by Rockström, Steffen [117]. It is, therefore, primarily useful if the climate footprint is of particular importance.

An open policy question is whether the external costs of climate change should fall under health budgets or be part of broader societal costs that ought to be accounted for by other payers (if by national bodies at all). In the latter case, it could be claimed that incorporating climate costs may lead to a misalignment with health system objectives, as it may divert resources from patient care to environmental concerns. However, this argument could be countered with reference to existing policies that also document that mitigating climate change is seen as a health system objective by a number of decision makers. Also, the idea that health is the sole objective of health systems could be accounted for by accounting for climate costs on the benefit side. Nevertheless, given that climate change mitigation at the expense of health gain is unlikely to find wide spread acceptance, climate-extended HEE is likely to be of particular health policy relevance if either funding for additional expenses to mitigate climate change is covered by other payers or if interventions can be identified that are climate-dominant (i.e., of similar conventional but more favorable climate-extended cost-effectiveness).

Along these lines, it could also be argued that alternative policy solutions like carbon pricing at a national level are more practical and, thus, preferable to accounting for the cost of carbon on a healthcare intervention level. Given the importance of Scope 2 emissions also in healthcare, for example, it is indeed reasonable to call for policies that promote the transition to regenerative energy on a national level. However, both national carbon prices and the impact of national energy policy are already accounted for in the non-traded price of carbon, and the requirement to consider geographical representativeness in these points is acknowledged.

At some point in the future, when (if?) all products face realistic pricing, adding climate impacts to HEE may no longer be necessary, so that this reporting framework becomes obsolete. However, given the current methodological work to extend the inclusion of external costs to more environmental impacts than CF alone, it appears likely that the methods to incorporate more external costs are developing at a higher pace than the policies to fully internalize them.

### Implications

Further work is necessary to provide more case studies on climate-extended HEE. Also, while this study provided general points to consider, a reporting standard that provides concise requirements for including climate impacts into HEE would be desirable to guide the work in this expanding field of research. For example, it would need to be determined whether providing a CF estimate with a published source is sufficient or whether transparent reporting requires extracting all methodological items from the cited studies (or explicitly stating if no information is available). Or, judgments need to be made about which information about different life cycle stages of the health technology subject to the evaluation ought to be provided within a climate-extended HEE. As this requires (mainly epistemic) value judgments, the development of such a reporting standard would benefit from using a formal Delphi procedure. Currently, such a standard is being developed under the acronym “CHEERS ClimatE” [118], and this study aims to provide the theoretical background to this Delphi procedure.

Given that not only the planetary boundary of climate change but that six of the nine planetary boundaries have already been transgressed [119], more work is needed on a more encompassing extension of CHEERS and HEE to cover all environmental impacts, even if there is no legal regulation to date. Using the working title CHEERS PlanetE, also a consensus process on such a reporting standard is currently in preparation [120] to provide guidance in circumstances where focusing on CF is less justified and when a result based on a single measure of environmental damage, like the ICFER, is less important. Depending on the requirements of these reporting standards, journal editors may need to reconsider the maximum word count of original studies in this field because information on CF and impacts integrated as costs or impacts adds to the information that needs to be reported in manuscripts.

Using health economic evidence in decision-making is already a challenging issue; therefore, information on ICFER, ICER-CCE, ICER-CEE, NMB-CEE, or NMB-CCE might not determine decision outcomes in the short term, at least unless incorporating carbon pricing becomes mandatory also for decision-makers using HEE. However, economic dominance is widely accepted. Therefore, we expect that as a first step, detecting ecologically dominant interventions (cf. climate-conscious prescription of inhaled medications of Schmiemann and Dörks [121]) may be the first task where climate-extended HEE has an impact to promote both more efficient and more climate-friendly decisions about new health technologies.

More policy-related work is needed to promote the inclusion of external effects related to climate change or to other planetary boundaries into the guidelines for HEE issued by healthcare payers so that work like the one reported here can pave the way towards more climate-friendly healthcare. A starting point would be a commonly agreed value or the conversion of climate damage into DALY or SC-CO_2_/non-traded price of carbon.

## Conclusion

While reporting frameworks of HEE have been extended to account for topics like equity or the value of research, how to transparently include external costs of environmental impacts like global warming is still an open topic. The methods for assessing environmental impacts are well developed, and core topics on how to account for them in HEE have been subject to vibrant scientific discussion. Now, there is a need for a structured standardization of analytic choices by decision makers and transparent reporting of analytic choices by the scientific community. This study provides a list of methodological points to consider for including climate impacts into HEE. Using CHEERS as a reference, it illustrates complementarities and connecting points between the methods of LCA and HEE and points towards analytic choices that need to be made and should be documented in a transparent manner. Further work is needed, such as a Delphi process to extend CHEERS towards climate impacts in an inclusive and transparent manner. Also, building an extension that incorporates other environmental effects or planetary boundaries other than GHGs would be desirable.

## Supplementary Information

Below is the link to the electronic supplementary material.

Supplementary Material 1 (DOCX 60.1 KB)

Supplementary Material 2 (DOCX 62.9 KB)

Supplementary Material 3 (DOCX 14.9 KB)

Supplementary Material 4 (DOCX 15.0 KB)

Supplementary Material 5 (DOCX 15.5 KB)

## Data Availability

Data is available upon request as an extra topic.

## Footnotes

1 https://www.england.nhs.uk/greenernhs/ [accessed Apr 14.^th^, 2025].

2 https://www.nice.org.uk/ [accessed Apr 14.^th^, 2025].

3 The fact that the impact is so much smaller on the effect side may also be due to the chosen value for the SC-CO_2_. If a lower price of €56 as recommended by CE Delft for the Netherlands ([116]) was chosen rather than €237 ([111]), the percentage changes of the results as consequence of incorporating CO_2_ damages as DALYS or as costs are more similar. Also, at this price, intervention B is dominated like in the other calculations (see the supplementary file Table 4 and 5).

## References

[1] Drummond, M.F., et al.: Methods for the economic evaluation of health care programmes. Oxford University Press. (2015)

[2] Cookson, R., et al.: Using cost-effectiveness analysis to address health equity concerns. Value Health **20**(2), 206–212 (2017) 28237196 10.1016/j.jval.2016.11.027 PMC5340318

[3] Husereau, D., et al.: Consolidated health economic evaluation reporting standards (CHEERS) statement. Int. J. Technol. Assess. Health Care **29**(2), 117–122 (2013) 23587340 10.1017/S0266462313000160

[4] Husereau, D., et al.: Consolidated health economic evaluation reporting standards 2022 (CHEERS 2022) statement: updated reporting guidance for health economic evaluations. Value Health **25**(1), 3–9 (2022) 35031096 10.1016/j.jval.2021.11.1351

[5] Husereau, D., et al.: Consolidated health economic evaluation reporting standards (CHEERS) 2022 explanation and elaboration: a report of the ISPOR CHEERS II good practices task force. Value Health **25**(1), 10–31 (2022) 35031088 10.1016/j.jval.2021.10.008

[6] Iglesias, C.P., et al.: Reporting guidelines for the use of expert judgement in model-based economic evaluations. Pharmacoeconomics **34**(11), 1161–1172 (2016) 27364887 10.1007/s40273-016-0425-9

[7] Kunst, N., et al.: Consolidated health economic evaluation reporting standards - value of information (CHEERS-VOI): explanation and elaboration. Value Health **26**(10), 1461–1473 (2023) 37414276 10.1016/j.jval.2023.06.014

[8] Lenzen, M., et al.: The environmental footprint of health care: a global assessment. The Lancet Planetary Health **4**(7), e271–e279 (2020) 32681898 10.1016/S2542-5196(20)30121-2

[9] Karliner, J.S., Scott; Boyd, Richard; Ashby, Ben; Steele, Kristian, Health Care´s Climate Footprint (Healthcare with no harm). Healthcare without harm. (2019). https://noharm-global.org/sites/default/files/documents-files/5961/HealthCaresClimateFootprint_092319.pdf. Accessed 22 Mar 2022

[10] Pichler, P.-P. et al.: Evidenzbasis* Treibhausgasemissionen des deutschen Gesundheitswesens - GermanHealthCFP*. Potsdam-Institut für Klimafolgenforschung (PIK) e.V. : Potsdam-Institut für Klimafolgenforschung (PIK) e.V. (2022). Sachbericht zum Projekt: Evidenzbasis Treibhausgasemissionen des deutschen Gesundheitswesens GermanHealthCFP. Accessed 12.2023

[11] World Health Organization.: Declaration of the seventh ministerial conference on environment and health. Budapest, Hungary. (2023)

[12] World Health Organization.: Final terms of reference of the alliance for transformative action on climate and health (ATACH). (2022). https://www.who.int/initiatives/alliance-for-transformative-action-on-climate-and-health/cop26-health-programme. Accessed 03.2022

[13] NHS England.: Suppliers - Net zero supplier roadmap. (2024). Available from: https://www.england.nhs.uk/greenernhs/get-involved/suppliers/. Accessed 29 Mar 2024

[14] Deutscher Bundestag.: Bundes-Klimaschutzgesetz (KSG) (2021)

[15] Hermann, A., Keimeyer, F.: Berücksichtgung von Klimaschutz- und Ressourcenschutzaspekten in der umweltfreundlichen öffentlichen Beschaffung - Darstellung der rechtlichen Lage (2024)

[16] Bobini, M., Cicchetti, A.: Integrating environmental sustainability into health technology assessment: an international survey of HTA stakeholders. Int. J. Technol. Assess. Health Care **40**(1), e64 (2024) 39610281 10.1017/S0266462324000631 PMC11703626

[17] Rogowski, W.: Accounting for planetary boundaries in health economic evaluation. Expert review of pharmaeconomics & outcomes research .(2024). 10.1080/14737167.2024.2364047 10.1080/14737167.2024.2364047 38904091

[18] Rogowski, W.H. and Elsner W.: How economics can help mitigate climate change - a critical review and conceptual analysis. Bremen papers on economics & innovation (2021). https://ideas.repec.org/p/atv/wpaper/2106.html

[19] Hensher, M.: Incorporating environmental impacts into the economic evaluation of health care systems: perspectives from ecological economics. Resour. Conserv. Recycl. (2020). 10.1016/j.resconrec.2019.104623

[20] Hensher, M.: Climate change, health and sustainable healthcare: the role of health economics. Health Econ **32**(5), 985–992 (2023) 36701185 10.1002/hec.4656

[21] Marsh, K., et al.: Expanding health technology assessments to include effects on the environment. Value Health **19**(2), 249–254 (2016) 27021760 10.1016/j.jval.2015.11.008

[22] Polisena, J., et al.: Environmental impact assessment of a health technology: a scoping review. Int. J. Technol. Assess. Health Care **34**(3), 317–326 (2018) 29897036 10.1017/S0266462318000351

[23] Pugliesi, P.-S., Marrauld, L., Lejeune, C.: Cost of carbon in the total cost of healthcare procedures: a methodological challenge. Appl. Health Econ. Health Policy **22**(5), 599–607 (2024) 38862769 10.1007/s40258-024-00890-4

[24] Greenwood Dufour, B., et al.: How we might further integrate considerations of environmental impact when assessing the value of health technologies. Int. J. Environ. Res. Public Health **19**(19), 12017 (2022) 36231319 10.3390/ijerph191912017 PMC9566650

[25] de Preux, L., Rizmie, D.: Beyond financial efficiency to support environmental sustainability in economic evaluations. Future Healthcare Journal **5**(2), 103 (2018) 31098543 10.7861/futurehosp.5-2-103 PMC6502566

[26] Ortsäter, G., et al.: Incorporating the environmental impact into a budget impact analysis: the example of adopting RESPIMAT® re-usable inhaler. Appl. Health Econ. Health Policy **18**, 433–442 (2020) 31808066 10.1007/s40258-019-00540-0 PMC7250803

[27] Ortsäter, G., et al.: A budget impact model to estimate the environmental impact of adopting RESPIMAT® re-usable in the Nordics and Benelux. Adv. Ther. **36**, 3435–3445 (2019) 31625130 10.1007/s12325-019-01114-1 PMC6860470

[28] Kponee-Shovein, K., et al.: Impact of choice of inhalers for asthma care on global carbon footprint and societal costs: a long-term economic evaluation. J. Med. Econ. **25**(1), 940–953 (2022) 35686860 10.1080/13696998.2022.2088196

[29] Kponee-Shovein, K., et al.: Carbon footprint and associated costs of asthma exacerbation care among UK adults. J. Med. Econ. **25**(1), 524–531 (2022) 35416088 10.1080/13696998.2022.2063603

[30] Debaveye, S., et al.: Human health benefit and burden of the schizophrenia health care pathway in Belgium: paliperidone palmitate long-acting injections. BMC Health Serv. Res. **19**, 1–16 (2019) 31217000 10.1186/s12913-019-4247-2 PMC6585029

[31] Debaveye, S., et al.: The public health benefit and burden of mass drug administration programs in Vietnamese schoolchildren: Impact of mebendazole. PLoS Negl. Trop. Dis. **12**(11), e0006954 (2018) 30419030 10.1371/journal.pntd.0006954 PMC6258429

[32] Kindred, M., Shabrina, Z., Zakiyah, N.: Exploratory approach to incorporating carbon footprint in health technology assessment (HTA) modelling: cost-effectiveness analysis of health interventions in the United Kingdom. Appl. Health Econ. Health Policy **22**(1), 49–60 (2024) 37948035 10.1007/s40258-023-00839-z PMC10761369

[33] Williams, J.T.W., et al.: Exploring the integration of environmental impacts in the cost analysis of the pilot MEL-SELF trial of patient-led melanoma surveillance. Appl. Health Econ. Health Policy **21**(1), 23–30 (2023) 36195819 10.1007/s40258-022-00765-6 PMC9834124

[34] Sanchez, S.A., Eckelman, M.J., Sherman, J.D.: Environmental and economic comparison of reusable and disposable blood pressure cuffs in multiple clinical settings. Resources, Conservation and Recycling **155**, 104643 (2020)

[35] Desterbecq, C., Tubeuf, S.: Inclusion of Environmental Spillovers in Applied Economic Evaluations of Healthcare Products. Value in Health **26**(8), 1270–1281 (2023) 36967027 10.1016/j.jval.2023.03.008

[36] Williams, J.T., et al.: Methods to include environmental impacts in health economic evaluations and health technology assessments: a scoping review. Value Health (2024). 10.1016/j.jval.2024.02.019 38462223 10.1016/j.jval.2024.02.019

[37] Guirado-Fuentes, C., et al.: Main challenges of incorporating environmental impacts in the economic evaluation of health technology assessment: a scoping review. Int. J. Environ. Res. Public Health **20**(6), 4949 (2023) 36981859 10.3390/ijerph20064949 PMC10049058

[38] Pinho-Gomes, A.-C., et al.: Incorporating environmental and sustainability considerations into health technology assessment and clinical and public health guidelines: a scoping review. Int. J. Technol. Assess. Health Care **38**(1), e84 (2022) 36510398 10.1017/S0266462322003282

[39] Carrandi, A., et al.: How environmental impact is considered in economic evaluations of critical care: a scoping review. Intensive Care Med. (1), 10 (2024). 10.1007/s00134-023-07274-7 10.1007/s00134-023-07274-7 PMC10810918 38191675

[40] Toolan, M., et al.: Environmental impact assessment in health technology assessment: principles, approaches, and challenges. Int. J. Technol. Assess. Health Care **39**(1), e13 (2023) 36815229 10.1017/S0266462323000041 PMC11570021

[41] McAlister, S., Morton, R.L., Barratt, A.: Incorporating carbon into health care: adding carbon emissions to health technology assessments. The Lancet Planetary Health **6**(12), e993–e999 (2022) 36495894 10.1016/S2542-5196(22)00258-3

[42] Walpole, S.C., et al.: How can environmental impacts be incorporated in health technology assessment, and how impactful would this be? Expert Rev. Pharmacoecon. Outcomes Res. (2023). 10.1080/14737167.2023.2248389 37578859 10.1080/14737167.2023.2248389

[43] Minotti, B., et al.: True cost accounting of a healthy and sustainable diet in Italy. Front. Nutr. **9**, 974768 (2022) 35967799 10.3389/fnut.2022.974768 PMC9372443

[44] Rey Aldana, D., et al.: Cost and potential savings of electronic consultation and its relationship with reduction in atmospheric pollution. Sustainability **13**(22), 12436 (2021)

[45] Marsh, K., et al.: Expanding health technology assessments to include effects on the environment. Value Health **19**(2), 249–254 (2016) 27021760 10.1016/j.jval.2015.11.008

[46] Ortsater, G., et al.: Incorporating the environmental impact into a budget impact analysis: the example of adopting RESPIMAT((R)) re-usable inhaler. Appl. Health Econ. Health Policy **18**(3), 433–442 (2020) 31808066 10.1007/s40258-019-00540-0 PMC7250803

[47] Ortsäter, G., et al.: A budget impact model to estimate the environmental impact of adopting RESPIMAT((R)) re-usable in the Nordics and Benelux. Adv. Ther. **36**(12), 3435–3445 (2019) 31625130 10.1007/s12325-019-01114-1 PMC6860470

[48] Aria, M., Cuccurullo, C.: Bibliometrix: an R-tool for comprehensive science mapping analysis. Journal of Informetrics **11**(4), 959–975 (2017)

[49] Owsianiak, M., et al.: LCA Applications. In: Hauschild, M.Z., Rosenbaum, R.K., Olsen, S.I. (eds.) Life cycle assessment. Springer (2018)

[50] DIN.: Environmental management - Life cycle assessment - Principles and framework (ISO 14040:2006 + Amd 1:2020) (2021)

[51] DIN.: Environmental management - Life cycle assessment - Requirements and guidelines (ISO 14044:2006 + Amd 1:2017 + Amd 2:2020) (2021)

[52] Wiedmann, T., Minx, J.: A definition of ‘carbon footprint.’ Ecol. Econ. Res. Trends **2008**(1), 1–11 (2008)

[53] International Organization for Standardization.: Greenhouse gases - Carbon footprint of products -Requirements and guidelines for quantification - DIN EN ISO 2018 14067 (2019)

[54] PAS 2050:2011.: Specification for the assessment of the life cycle greenhouse gas emissions of goods and services. Bsi Br. Stand. Isbn, **978**, 580 (2008)

[55] WBCSD, W.: A corporate accounting and reporting standard*.* World resources institute and world business council for sustainable development (2004)

[56] Garcia, R., Freire, F.: Carbon footprint of particleboard: a comparison between ISO/TS 14067, GHG protocol, PAS 2050 and climate declaration. Journal of Cleaner Production **66**, 199–209 (2014)

[57] Lange, O., et al.: A transparency checklist for carbon footprint calculations applied within a systematic review of virtual care interventions. Int. J. Environ. Res. Public Health **19**(7474), 1–14 (2022) 10.3390/ijerph19127474 PMC9223517 35742724

[58] Frischknecht, R., et al.: The ecoinvent database: overview and methodological framework. Int. J. Life Cycle Assess. **10**(1), 3–9 (2005)

[59] Nicolet, J., et al.: What is the carbon footprint of primary care practices? A retrospective life-cycle analysis in Switzerland. Environ. Health **21**(1), 1–10 (2022) 34980135 10.1186/s12940-021-00814-y PMC8723904

[60] Smith, C.L., Zurynski, Y., Braithwaite, J.: We can’t mitigate what we don’t monitor: using informatics to measure and improve healthcare systems’ climate impact and environmental footprint. J. Am. Med. Inform. Assoc. **29**(12), 2168–2173 (2022) 35822400 10.1093/jamia/ocac113 PMC9667189

[61] Stadler, K., Wood, R., Bulavskaya, T., Södersten, C.-J., Simas, M., Schmidt, S., Usubiaga, A., Acosta-Fernández, J., Kuenen, J., Bruckner, M., Giljum, S., Lutter, S., Merciai, S., Schmidt, J. H., Theurl, M. C., Plutzar, C., Kastner, T., Eisenmenger, N., Erb, K.-H., . . . Tukker, A.: EXIOBASE 3: Developing a time series of detailed environmentally extended multi-regional input-output tables*.* J. Ind. Ecol. **22**(3), 502–515 (2018)

[62] Steubing, B., et al.: How do carbon footprints from LCA and EEIOA databases compare? A comparison of ecoinvent and EXIOBASE. J. Ind. Ecol. **26**(4), 1406–1422 (2022)

[63] Schaubroeck, T.: Relevance of attributional and consequential life cycle assessment for society and decision support. Front. Sustain. (2023). 10.3389/frsus.2023.1063583

[64] McAlister, S., et al.: How many carbon emissions are saved by doing one less MRI? The Lancet Planetary Health **8**(6), e350–e350 (2024) 38849176 10.1016/S2542-5196(24)00092-5

[65] Suh, S., Huppes, G.: Methods for life cycle inventory of a product. J. Clean. Prod. **13**(7), 687–697 (2005)

[66] Pomponi, F., Lenzen, M.: Hybrid life cycle assessment (LCA) will likely yield more accurate results than process-based LCA. J. Clean. Prod. **176**, 210–215 (2018)

[67] Eckelman, M.J., Sherman, J.D., MacNeill, A.J.: Life cycle environmental emissions and health damages from the Canadian healthcare system: an economic-environmental-epidemiological analysis. PLoS Med **15**(7), e1002623 (2018) 30063712 10.1371/journal.pmed.1002623 PMC6067712

[68] Kazi, D.S., et al.: Climate change and cardiovascular health: a systematic review. JAMA Cardiol **9**(8), 748–757 (2024) 38865135 10.1001/jamacardio.2024.1321 PMC11366109

[69] Chevance, G., et al.: Digital health at the age of the Anthropocene. The Lancet Digital Health **2**(6), E290–E291 (2020) 33328121 10.1016/S2589-7500(20)30130-8 PMC7928047

[70] Malhi, J.K., et al.: Climate change and cardiovascular health: Recent updates and actions for healthcare. American Heart Journal Plus: Cardiology Research and Practice **45**, 100443 (2024) 39246679 10.1016/j.ahjo.2024.100443 PMC11377132

[71] Lokmic-Tomkins, Z., et al.: Assessing the carbon footprint of digital health interventions: a scoping review. J. Am. Med. Inform. Assoc. **29**(12), 2128–2139 (2022) 36314391 10.1093/jamia/ocac196 PMC9667173

[72] Lange, O., et al.: A transparency checklist for carbon footprint calculations applied within a systematic review of virtual care interventions. Int. J. Environ. Res. Public Health **19**(12), 7474 (2022) 35742724 10.3390/ijerph19127474 PMC9223517

[73] Smith, A.J., et al.: The carbon footprint of behavioural support services for smoking cessation. Tob. Control. **22**(5), 302–307 (2013) 23481905 10.1136/tobaccocontrol-2012-050672

[74] International Organization for Standardization.: Environmental management-Life cycle assessment -requirements and guidelines - DIN EN ISO 14044 (2018)

[75] Drummond, M.F., et al.: Methods for the economic evaluation of health care programmes. Fourth edition ed. Oxford University Press, Oxford New York (2015)

[76] European Commission.: International Reference Life Cycle Data System (ILCD) Handbook- General guide for Life Cycle Assessment, Joint Research Centre - Institute for Environment and Sustainability: Luxembourg (2010)

[77] McGain, F., et al.: Financial and environmental costs of reusable and single-use anaesthetic equipment. Br. J. Anaesth. **118**(6), 862–869 (2017) 28505289 10.1093/bja/aex098

[78] International Organization for Standardization.: Environmental management - Life cycle assessment - Principles and framework -DIN EN ISO 14040 (2006)

[79] IPCC, et al.: Climate Change 2013: The physical science basis. In: Contribution of Working Group I to the Fifth Assessment Report of the Intergovernmental Panel on Climate Change in Cambridge University Press. p 1535 (2013)

[80] Myhre, G.D., et al.: Anthropogenic and Natural Radiative Forcing. In: Stocker, T.F. et al. (eds) Climate Change 2013: The physical science basis. Contribution of Working Group I to the Fifth Assessment Report of the Intergovernmental Panel on Climate Change. Cambridge University Press (2013)

[81] Lueddeckens, S., Saling, P., Guenther, E.: Temporal issues in life cycle assessment—a systematic review. Int. J. Life Cycle Assess **25**, 1385–1401 (2020)

[82] Lueddeckens, S., Saling, P., Guenther, E.: Discounting and life cycle assessment: a distorting measure in assessments, a reasonable instrument for decisions. Int. J. Environ. Sci. Technol. (2021). 10.1007/s13762-021-03426-8

[83] Pehnt, M.: Dynamic life cycle assessment (LCA) of renewable energy technologies. Renewable Energy **31**(1), 55–71 (2006)

[84] Hellweg, S., Hofstetter, T.B., Hungerbuhler, K.: Discounting and the environment should current impacts be weighted differently than impacts harming future generations? Int. J. Life Cycle Assess. **8**, 8–18 (2003)

[85] Huijbregts, M.A., et al.: ReCiPe 2016: a harmonized life cycle impact assessment method at midpoint and endpoint level report I: characterization (2016)

[86] Goedkoop, M.J.: The Eco-indicator 99 a damage oriented method for life cycle impact assessment methodology report. Pre Concultants (1999)

[87] Bulle, C., et al.: IMPACT world+: a globally regionalized life cycle impact assessment method. Int. J. Life Cycle Assess. **24**(9), 1653–1674 (2019) 10.1007/s11367-018-1539-4 PMC7592718 33122880

[88] Verones, F., et al.: Lc-impact: a regionalized life cycle damage assessment method. J. Ind. Ecol. **24**(6), 1201–1219 (2020)

[89] Institute for Health Metrics and Evaluation (IHME).: Global burden of disease 2021: Findings form the GBD 2021 Study (2024)

[90] Neumann, P.J., et al.: Comparing the cost-per-QALYs gained and cost-per-DALYs averted literatures. Gates Open Res. **2**, 5 (2018) 29431169 10.12688/gatesopenres.12786.1 PMC5801595

[91] Rennert, K., et al.: Comprehensive evidence implies a higher social cost of CO2. Nature **610**(7933), 687–692 (2022) 36049503 10.1038/s41586-022-05224-9 PMC9605864

[92] International Organization for Standardization.: Environmental management - Guidelines for determining environmental costs and benefits in ISO 14007:2019 (2019)

[93] International Organization for Standardization.: ISO 14008 Monetary valuation of environmental impacts and related environmental aspects -DIN EN ISO 14008:2019(E) (2019)

[94] Tol, R.S.: Social cost of carbon estimates have increased over time. Nat. Clim. Chang. **13**(6), 532–536 (2023)

[95] Wang, P., et al.: Estimates of the social cost of carbon: a review based on meta-analysis. J. Clean. Prod. **209**, 1494–1507 (2019)

[96] Caesary, D., Kim, H., Nam, M.J.: An alternative approach to capture uncertainties embedded in the estimation of social cost of carbon. WIREs Energy Environ. **12**(4), e475 (2023)

[97] Umweltbundesamt.: *Gesellschaftliche Kosten von Umweltbelastungen* **2023**(21.08.) (2023). https://www.umweltbundesamt.de/daten/umwelt-wirtschaft/gesellschaftliche-kosten-von-umweltbelastungen#gesamtwirtschaftliche-bedeutung-der-umweltkosten. Accessed 12.2024

[98] IPCC, et al.: Climate change 2022 mitigation of climate change - Working Group III Contribution to the Sixth Assessment Report of the Intergovernmental Panel on Climate Change - Summary for policymakers (2022)

[99] Shindell, D., Fuglestvedt, J., Collins, W.: The social cost of methane: theory and applications. Faraday Discuss **200**, 429–451 (2017) 28581559 10.1039/c7fd00009j

[100] Shindell, D.T.: The social cost of atmospheric release. Clim. Change **130**, 313–326 (2015)

[101] Narassimhan, E., et al.: Carbon pricing in practice: a review of existing emissions trading systems. Clim. Pol. **18**(8), 967–991 (2018)

[102] UNFCCC.: Kyoto Protocol to th United Nations Framework Convention on Climate Change (1997)

[103] Intergovernmental Panel on Climate Change.: Emission factor database (EFDB). (2023)

[104] Goedkoop, M., et al.: ReCiPe 2008*.* A life cycle impact assessment method which comprises harmonised category indicators at the midpoint and the endpoint level. **1**, 1-126 (2009)

[105] Weidema, B.P., Wesnæs, M.S.: Data quality management for life cycle inventories—an example of using data quality indicators. J. Clean. Prod. **4**(3–4), 167–174 (1996)

[106] Keil, M., et al.: The impact of switching from single-use to reusable healthcare products: a transparency checklist and systematic review of life-cycle assessments. Eur. J. Public Health **33**(1), 56–63 (2023) 36433787 10.1093/eurpub/ckac174 PMC9898010

[107] Clay, N., et al.: What is the climate footprint of therapeutic diets for people with chronic kidney disease? Results from an Australian analysis. J. Hum. Nutr. Diet. **36**(6), 2246–2255 (2023) 37427492 10.1111/jhn.13204

[108] iPoint-systems gmbh. Life Cycle Impact Assessment- the most widely used LCIA Methods. (2018). Available from: https://go.ipoint-systems.com/blog/lcia-indicator. Accessed 13 Sept 2024

[109] van den Bergh, J.C., Botzen, W.: Monetary valuation of the social cost of CO2 emissions: a critical survey. Ecol. Econ. **114**, 33–46 (2015)

[110] Fraas, A.G., et al.: Seven recommendations for pricing greenhouse gas emissions. Journal of Benefit-Cost Analysis **14**(2), 191–204 (2023)

[111] Matthey, A., Bünger, B.: *Methodenkonvention 3.1 zur Ermittlung von Umweltkosten Kostensätze Stand 12/2020*. Umweltbundesamt: Dessau-Roßlau (2020)

[112] Shukla, P.R., et al.: Climate Change 2022 Mitigation of Climate Change. Working Group III Contribution to the Sixth Assessment Report of the Intergovernmental Panel on Climate Change. Summary for Policymakers. [IPCC]: [Geneva] (2022)

[113] Schwarzer, R., et al.: Systematic overview of cost–effectiveness thresholds in ten countries across four continents. J. Comp. Eff. Res. **4**(5), 485–504 (2015) 26490020 10.2217/cer.15.38

[114] Grosse, S.D.: Assessing cost-effectiveness in healthcare: history of the $50,000 per QALY threshold. Expert Rev. Pharmacoecon. Outcomes Res. **8**(2), 165–178 (2008) 20528406 10.1586/14737167.8.2.165

[115] Rogowski, W., John, J.: Preferences as fairness judgments: a critical review of normative frameworks of preference elicitation and development of an alternative based on constitutional economics. Cost Eff. Resour. Alloc. **22**(1), 10 (2024) 38291472 10.1186/s12962-024-00510-x PMC10826070

[116] de Bruyn, S.: Environmental Prices Handbook EU 28 version. CE Delft, Delft (2018)

[117] Rockström, J., et al.: A safe operating space for humanity. Nature **461**(7263), 472–475 (2009) 19779433 10.1038/461472a

[118] Oldenburg, J., et al.: Adding climate costs to health economic evaluation - the Cheers Climate checklist. Registered at Open Science Framework. (2023). https://bmjopen.bmj.com/content/15/9/e094617.abstract

[119] Richardson, K., et al.: Earth beyond six of nine planetary boundaries. Sci. Adv. **9**(37), eadh2458 (2023) 37703365 10.1126/sciadv.adh2458 PMC10499318

[120] Oldenburg, J., et al.: Accounting for planetary boundaries in health economic evaluation: the CHEERS PlanetE checklist. Registered at Open Science Framework. (2023)

[121] Schmiemann, G. and M. Dörks, Climate-conscious prescription of inhaled medications: DEGAM S1-Guideline. (2022) 10.1055/a-2561-9329 40359985

