# Supplementary material for: Points to consider for incorporating climate impacts into health economic evaluation
Source: Eur J Health Econ. 2026 Jan 7;27(6):1261–76. doi: 10.1007/s10198-025-01859-3 (PMC13451359; doi:10.1007/s10198-025-01859-3)
Supplement: Supplementary file 1 [file 10198_2025_1859_MOESM1_ESM.docx]

R-Code-Pubmed-Search.R

Oldenburg

2025-04-29

```
#Searchstrategy:
#"health economic evaluation"[Title/Abstract] OR "health technology assessment*"[Title/Abstract ]
#AND ("climate"[Title/Abstract] OR "environment* "[Title/Abstract] OR "carbon*"[Title/Abstract] OR "greenhouse gas*"[Title/Abstract])
#AND ("integrat*"[Title/Abstract] OR "inclu*"[Title/Abstract] OR "consider*"[Title/Abstract] OR "Expand*"[Title/Abstract] OR "Incorporat*"[Title/Abstract] OR "extend*"[title/abstract]))

#Packages
# CRAN-Spiegel festlegen (Deutschland)
options(repos = c(CRAN = "https://cran.r-project.org"))# CRAN-Spiegel festlegen (Deutschland)
install.packages("bibliometrix")
library(bibliometrix)
install.packages("pubmedR")
library(pubmedR)
library(ggplot2)
library(dplyr)
install.packages("officer")
library(officer)
install.packages("flextable")
library(flextable)
library(magrittr)


# Datei einlesen
file_path <- "C:\\Users\\Oldenburg\\SynologyDrive\\Literatur\\Manteltext\\Literatursuche\\pubmed-healthecon-set(5).txt"
pubmed_data <- convert2df(file_path, dbsource = "pubmed", format = "pubmed")
# Datenübersicht
head(pubmed_data)
# Schlüsselbegriffe für die Suchstrategie
main_terms <- "(health economic evaluation|health technology assessment)"
climate_terms <- "(climate|environment|carbon|greenhouse gas)"
integration_terms <- "(integrat|inclu|consider|expand|incorporat|extend)"

# Kombinierte Filter anwenden
filtered_data <- pubmed_data[
  grepl(main_terms, pubmed_data$TI, ignore.case = TRUE) |
    grepl(main_terms, pubmed_data$AB, ignore.case = TRUE) &
    (grepl(climate_terms, pubmed_data$TI, ignore.case = TRUE) |
       grepl(climate_terms, pubmed_data$AB, ignore.case = TRUE)) &
    (grepl(integration_terms, pubmed_data$TI, ignore.case = TRUE) |
       grepl(integration_terms, pubmed_data$AB, ignore.case = TRUE)), ]

head(filtered_data[, c("TI","AB","AU")])
# Veröffentlichungsjahr als numerischen Wert konvertieren
filtered_data$Year <- as.numeric(filtered_data$PY)

# Zähle Artikel pro Jahr
yearly_counts <- table(filtered_data$Year)

# Datenrahmen erstellen
df_yearly <- data.frame(
  Year = as.numeric(names(yearly_counts)),
  Count = as.integer(yearly_counts)
)

ggplot(df_yearly, aes(x = Year, y = Count)) +
  geom_line(color = "black") +
  geom_point(color = "black") +
  geom_smooth(method = "loess", se = FALSE, color = "blue", size = 1.2) +
  labs(title = "Number of publication per year",
       x = "Year", y = "Number of articles") +
  theme_minimal()
## Warning: Using `size` aesthetic for lines was deprecated in ggplot2 3.4.0.
## ℹ Please use `linewidth` instead.
## This warning is displayed once every 8 hours.
## Call `lifecycle::last_lifecycle_warnings()` to see where this warning was
## generated.
## `geom_smooth()` using formula = 'y ~ x'
```

```
ggplot(df_yearly, aes(x = Year, y = Count)) +
  geom_smooth(method = "loess", se = FALSE, color = "blue", size = 1.2) +
  labs(title = "Number of publication per year",
       x = "Year", y = "Number of articles") +
  theme_minimal()
## `geom_smooth()` using formula = 'y ~ x'
```

```
doc <- read_docx()
doc <- body_add_par(doc, "Anzahl der Veröffentlichungen pro Jahr", style = "heading 1")
doc <- body_add_flextable(doc, flextable(df_yearly))
doc <- body_add_par(doc, "Quelle: Eigene Analyse", style = "Normal")

print(doc, target = "veroeffentlichungen_pro_jahr.docx")

# Dokument speichern
print(doc, target = "veroeffentlichungen_pro_jahr.docx")


library(wordcloud)
## Lade nötiges Paket: RColorBrewer
# Titel als Text zusammenfassen
all_titles <- tolower(paste(filtered_data$TI, collapse = " "))
all_titles <- gsub("[^\\w\\s]", "", all_titles)


stopwords <- c("and", "or", "of", "the", "a", "in", "for", "to", "on",
               "with", "by", "at", "an", "as", "is", "are", "be", "this",
               "that", "was", "were", "has", "have", "not", "but", "if",
               "from")

# Wörter extrahieren
words <- unlist(strsplit(all_titles, "\\s+"))
word_freq <- table(words)
# Entferne Stopwörter
words <- words[!words %in% stopwords]

# Häufigkeit der Wörter berechnen
word_freq <- table(words)

# Wörter hervorheben
highlight_words <- c("environment","feasibility","impact")  # Wörter, die hervorgehoben werden sollen
colors <- ifelse(names(word_freq) %in% highlight_words, "red", "black")

# Wortwolke mit farblichen Hervorhebungen
wordcloud(names(word_freq), freq = word_freq, max.words = 100, random.order = FALSE, colors = colors)
# Wortwolke erstellen
wordcloud(names(word_freq), freq = word_freq, max.words = 100, random.order = FALSE)
# Prüfen, welche Spalten im Datensatz enthalten sind
colnames(pubmed_data)
##  [1] "AU"       "AF"       "DE"       "AID"      "OT"       "PHST"
##  [7] "DT"       "AB"       "C1"       "OI"       "BTI"      "CI"
## [13] "CIN"      "CN"       "COIS"     "CON"      "CRDT"     "CTI"
## [19] "DCOM"     "DEP"      "PY"       "EDAT"     "EIN"      "FIR"
## [25] "GR"       "IS"       "IR"       "IRAD"     "SN"       "ISBN"
## [31] "JID"      "SO"       "LA"       "LID"      "LR"       "MHDA"
## [37] "MID"      "OAB"      "OABL"     "OID"      "OTO"      "OWN"
## [43] "PB"       "PP"       "PL"       "PMC"      "PMCR"     "PMID"
## [49] "PST"      "RF"       "RN"       "SB"       "SI"       "SO2"
## [55] "STAT"     "J9"       "TI"       "TT"       "UIN"      "UOF"
## [61] "VL"       "DI"       "DB"       "ID"       "RP"       "TC"
## [67] "CR"       "AU_UN"    "AU1_UN"   "AU_UN_NR" "SR_FULL"  "SR"
# Vorschau der relevanten Spalten (z. B. C1 für Adressen)
head(pubmed_data$PL)
## [1] "ENGLAND"       "NETHERLANDS"   "ENGLAND"       "SWITZERLAND"
## [5] "UNITED STATES" "ENGLAND"
# Extrahiere Adressen
countries <- pubmed_data$PL
countries <- trimws(countries)  # Entfernt Leerzeichen
countries <- countries[!is.na(countries)]  # Entfernt NA-Werte

# Häufigkeit der Länder berechnen
country_counts <- table(countries)

# Zeige die Häufigkeiten
print(country_counts)
## countries
##           BRAZIL           CANADA          ENGLAND          GERMANY
##                1               16              122                6
##           GREECE            INDIA             IRAN          IRELAND
##                1                1                2                4
##            ITALY            JAPAN           LONDON      NETHERLANDS
##                2                1                1                9
##      NEW ZEALAND     OSLO, NORWAY      OTTAWA (ON)   ROCKVILLE (MD)
##               14                1                5                1
## SOUTHAMPTON (UK)      SWITZERLAND    UNITED STATES
##                2               22               34
# Häufigkeiten als DataFrame für Visualisierung
df_countries <- data.frame(
  Country = names(country_counts),
  Count = as.integer(country_counts)
)

# Extrahiere Länder aus den Adressen
library(stringr)
##
## Attache Paket: 'stringr'
## Das folgende Objekt ist maskiert durch '.GlobalEnv':
##
##     words
# Extrahiere die Spalte PL (Publikationsland/Stadt)
addresses <- pubmed_data$PL

# Mapping von Städten zu Ländern
city_to_country <- c(
  "OTTAWA (ON)" = "CANADA",
  "LONDON" = "ENGLAND",
  "SOUTHAMPTON (UK)" = "ENGLAND",
  "ROCKVILLE (MD)" = "UNITED STATES",
  "OSLO, NORWAY"="NORWAY"
)

# Städte in Ländern umwandeln (wenn Adresse eine Stadt ist, zugeordnetes Land verwenden)
countries <- ifelse(addresses %in% names(city_to_country),
                    city_to_country[addresses],  # Wenn Stadt, dann Land aus Mapping
                    addresses)  # Andernfalls das Original behalten

# Bereinigung: Entferne Leerzeichen und NA-Werte
countries <- trimws(countries)
countries <- countries[!is.na(countries)]

# Häufigkeiten der Länder berechnen
country_counts <- table(countries)

# Häufigkeiten als DataFrame für Visualisierung
df_countries <- data.frame(
  Country = names(country_counts),
  Count = as.integer(country_counts)
)
df_countries$Country <- str_to_title(df_countries$Country)

# Vorschau der Länder-Häufigkeiten
print(df_countries)
##          Country Count
## 1         Brazil     1
## 2         Canada    21
## 3        England   125
## 4        Germany     6
## 5         Greece     1
## 6          India     1
## 7           Iran     2
## 8        Ireland     4
## 9          Italy     2
## 10         Japan     1
## 11   Netherlands     9
## 12   New Zealand    14
## 13        Norway     1
## 14   Switzerland    22
## 15 United States    35
# Visualisierung: Balkendiagramm der Länder
ggplot(df_countries, aes(x = reorder(Country, -Count), y = Count)) +
  geom_bar(stat = "identity", fill = "skyblue") +
  coord_flip() +
  labs(title = "Amount of articles published by location",
       x = "countries", y = "number of articles") +
  theme_minimal()
```

```
# Nach Ort der Autoren
head (pubmed_data$C1,10)
# Die Spalte "C1" enthält die Adressen
addresses2 <- pubmed_data$C1

# Extrahiere das letzte Wort in Großbuchstaben, das das Land sein könnte
extracted_countries <- str_extract(addresses2, "\\b[A-Z]+\\b(?=\\.;)")

# Entferne NA-Werte, die bei leeren Einträgen auftreten können
extracted_countries <- extracted_countries[!is.na(extracted_countries)]

# Vorschau der extrahierten Länder
print(extracted_countries)
##   [1] "GERMANY"         "DEUTSCHLAND"     "INC"             "ITALY"
##   [5] "SWEDEN"          "UK"              "BELGIUM"         "CANADA"
##   [9] "JAPAN"           "NETHERLANDS"     "GREECE"          "UK"
##  [13] "IRAN"            "F"               "ITALY"           "UK"
##  [17] "UK"              "UK"              "GERMANY"         "GERMANY"
##  [21] "FRANCE"          "INC"             "EMIRATES"        "BELGIUM"
##  [25] "CANADA"          "AUSTRALIA"       "ITALY"           "AUSTRALIA"
##  [29] "CANADA"          "SPAIN"           "SPAIN"           "GEBBERTSTR"
##  [33] "NETHERLANDS"     "CANADA"          "UK"              "UK"
##  [37] "TAIWAN"          "CANADA"          "UK"              "T"
##  [41] "SWEDEN"          "STATES"          "USA"             "INC"
##  [45] "EMIRATES"        "UK"              "BELGIUM"         "UK"
##  [49] "AV"              "CHINA"           "HUNGARY"         "SPAIN"
##  [53] "UK"              "AUSTRALIA"       "UK"              "SPAIN"
##  [57] "DEPT"            "AUSTRALIA"       "ITALY"           "UK"
##  [61] "AUSTRALIA"       "BULGARIA"        "CANADA"          "CROATIA"
##  [65] "ITALY"           "EMILY"           "ITALY"           "SHEFFIELD"
##  [69] "UK"              "CANADA"          "GERMANY"         "AUSTRALIA"
##  [73] "CANADA"          "UK"              "UK"              "INDIA"
##  [77] "IRAN"            "AUSTRALIA"       "UK"              "UK"
##  [81] "UK"              "UK"              "UK"              "KINGDOM"
##  [85] "CANADA"          "SWITZERLAND"     "UK"              "M"
##  [89] "AUSTRALIA"       "KINGDOM"         "GERMANY"         "CANADA"
##  [93] "UK"              "UK"              "UK"              "UK"
##  [97] "SWEDEN"          "CANADA"          "SPAIN"           "BELGIUM"
## [101] "CANADA"          "ST"              "UK"              "KINGDOM"
## [105] "KINGDOM"         "BELGIUM"         "UK"              "UK"
## [109] "UK"              "UK"              "UK"              "GREGOR"
## [113] "UK"              "BRITAIN"         "UK"              "STR"
## [117] "UK"              "UK"              "FRANCISCO"       "EVIDENCEHERBERT"
## [121] "CADTH"           "CADTH"           "UK"              "LATVIA"
## [125] "CROATIA"         "COMMITTEE"       "UK"              "CANADA"
## [129] "KINGDOM"         "UK"              "ITALY"           "UK"
## [133] "UK"              "UK"              "AFRICA"          "EOPYY"
## [137] "KOREA"           "UK"              "UK"              "KINGDOM"
## [141] "UK"              "INC"             "UK"              "SV"
## [145] "EVIDERAKEVIN"    "LTD"             "IHE"             "BARCELONA"
## [149] "SWITZERLAND"     "UK"              "UK"              "UK"
## [153] "DD"              "UK"              "QC"              "I"
## [157] "GERMANY"         "CANADA"          "UK"              "INDIA"
## [161] "GERMANY"         "UK"              "UK"              "CANADA"
## [165] "SCIENCE"         "KINGDOM"         "CALIFORNIA"      "UK"
## [169] "UK"              "BRASIL"          "UK"              "UK"
## [173] "EGYPT"           "CANADA"          "SWEDEN"          "ARGENTINA"
## [177] "SWEDEN"          "USA"             "GERMANY"         "BELGIUM"
## [181] "ITALY"           "UK"              "UK"              "ROMA"
## [185] "KOREA"           "UK"              "CANADA"          "K"
## [189] "BC"              "CANADA"          "C"               "CANADA"
## [193] "UK"              "NETHERLANDS"     "GERMANY"         "UK"
## [197] "DEUTSCHLAND"     "CANADA"          "FINLAND"         "SWEDEN"
## [201] "HEALTH"          "GERMANY"         "KBV"             "AFRICA"
## [205] "UNIVERSITY"      "ITALY"           "CANADA"          "SWITZERLAND"
## [209] "UK"              "AUSTRALIA"
valid_countries <- c(
  "UK", "USA", "UNITED KINGDOM", "ENGLAND", "UNITED STATES",
  "AUSTRALIA", "NEW ZEALAND", "GERMANY", "IRELAND",
  "NETHERLANDS", "CANADA", "ITALY", "SWITZERLAND", "IRAN",
  "GREECE", "INDIA", "NORWAY", "FRANCE", "BELGIUM", "DENMARK",
  "SWEDEN", "POLAND", "SPAIN", "PORTUGAL", "ARGENTINA", "KOREA",
  "EGYPT", "AFRICA", "CHINA", "HUNGARY", "CROATIA", "MALAYSIA","DEUTSCHLAND","LATVIA","BARCELONA","EMIRATES","TAIWAN","BULGARIA","SHEFFIELD","KINGDOM","BRITAIN","NICE","NEW ZEALAND"
)

city_to_country2 <- c(
  "BARCELONA" = "SPAIN",
  "DEUTSCHLAND" = "GERMANY",
  "KINGDOM" = "UK",
  "BRITAIN" = "UK",
  "NICE"="UK",
  "SHEFFIELD"= "UK"
)

# Schritt 1: Städte durch Länder ersetzen, falls sie in city_to_country2 vorhanden sind
adjusted_countries <- ifelse(
  extracted_countries %in% names(city_to_country2),
  city_to_country2[extracted_countries],  # Ersetze durch das zugehörige Land
  extracted_countries                     # Behalte den Originalwert
)

# Schritt 2: Filtere nur gültige Länder aus adjusted_countries
filtered_countries2 <- adjusted_countries[adjusted_countries %in% valid_countries]

# Schritt 3: Häufigkeit der Länder berechnen
country_counts2 <- table(filtered_countries2)

# Schritt 4: Umwandlung in DataFrame für Visualisierung
df_countries2 <- data.frame(
  Country = names(country_counts2),
  Count = as.integer(country_counts2)
)
print(df_countries2)
##        Country Count
## 1       AFRICA     2
## 2    ARGENTINA     1
## 3    AUSTRALIA     9
## 4      BELGIUM     6
## 5     BULGARIA     1
## 6       CANADA    21
## 7        CHINA     1
## 8      CROATIA     2
## 9        EGYPT     1
## 10    EMIRATES     2
## 11      FRANCE     1
## 12     GERMANY    12
## 13      GREECE     1
## 14     HUNGARY     1
## 15       INDIA     2
## 16        IRAN     2
## 17       ITALY     9
## 18       KOREA     2
## 19      LATVIA     1
## 20 NETHERLANDS     3
## 21       SPAIN     6
## 22      SWEDEN     6
## 23 SWITZERLAND     3
## 24      TAIWAN     1
## 25          UK    72
## 26         USA     2
df_countries2 <- df_countries2[df_countries2$Country != "AFRICA", ]

df_countries3 <- df_countries2[order(-df_countries2$Count), ][1:5, ]

df_countries3$Country <- str_to_title(tolower(df_countries3$Country))
df_countries3$Country <- ifelse(df_countries3$Country == "Uk",
                                "United Kingdom",
                                df_countries3$Country)


# Schritt 5: Visualisierung der Länderhäufigkeiten
ggplot(df_countries3, aes(x = reorder(Country, -Count), y = Count)) +
  geom_bar(stat = "identity", fill = "skyblue") +
  coord_flip() +
  labs(title = "Top 5 countries by number of articles",
       x = "country", y = "number of article") +
  theme_minimal()
```

```
# Prozentuale Anteile berechnen
df_countries3$Percentage <- round((df_countries3$Count / sum(df_countries3$Count)) * 100, 1)

# Korrekte Position der Beschriftungen berechnen
df_countries3 <- df_countries3 %>%
  arrange(desc(Country)) %>%  # Sortierung nach Country für konsistentes Layout
  mutate(Position = cumsum(Count) - (Count / 2))  # Mittelposition für jedes Kuchenstück

# Vorschau des DataFrame mit Position
print(df_countries3)
##          Country Count Percentage Position
## 1 United Kingdom    72       58.5     36.0
## 2          Italy     9        7.3     76.5
## 3        Germany    12        9.8     87.0
## 4         Canada    21       17.1    103.5
## 5      Australia     9        7.3    118.5
# Kreisdiagramm mit korrekt positionierten Prozenten
ggplot(df_countries3, aes(x = "", y = Count, fill = Country)) +
  geom_bar(stat = "identity", width = 1, color = "white") +  # Kuchenstücke erstellen
  coord_polar("y", start = 0) +  # Konvertiere zu einem Kreisdiagramm
  geom_text(aes(label = paste0(Percentage, "%")),
            position = position_stack(vjust = 0.5),  # Positioniere die Prozentwerte in der Mitte
            size = 3, color = "white") +  # Textgröße und Farbe
  labs(title = "Top 10 countries by number of articles",
       x = NULL, y = NULL, fill = "Country") +
  theme_void() +  # Entferne unnötige Achsen und Hintergründe
  theme(plot.title = element_text(hjust = 0.5))
```

```
library(treemap)

treemap(
  df_countries3,
  index = "Country",
  vSize = "Count",
  type = "index",
  title = "Top 10 countries by number of articles (authors' affiliation)"
)
```
