# Supplementary material for: Points to consider for incorporating climate impacts into health economic evaluation
Source: Eur J Health Econ. 2026 Jan 7;27(6):1261–76. doi: 10.1007/s10198-025-01859-3 (PMC13451359; doi:10.1007/s10198-025-01859-3)
Supplement: Supplementary file 2 [file 10198_2025_1859_MOESM2_ESM.docx]

Points to consider for reporting climate impacts alongside a HEE, illustrated by a hypothetical case study

| **#** | **Section / Topic** | Guidance for reporting | **Climate extension?** | **Points to consider for reporting climate impacts alongside a HEE** | **Exemplary application within a hypothetical public health intervention** |
| --- | --- | --- | --- | --- | --- |
|  | Explanation of column | Original CHEERS Item | Were specific methodological points to report were identified? (no/ optional / yes) | This column explains connections between methods of economic evaluation and climate footprint (CF), including items from existing CF guidelines and why they are relevant. | This column contains an example of how climate impacts could be integrated in HEE, using four hypothetical case study interventions for preventing diabetes in individuals with obesity: A- health promotion with a focus on plant-based diet, B – intensive nutritional counselling for planetary health diet, C- CO_2_ intensive formula diet, D- psychotherapy aimed at behavioural change towards a low carb diet. |
|  | **Title** |  |  |  |  |
| **1** | Title | Identify the study as an economic evaluation and specify the interventions being compared. | Yes | Studies should be identifiable through database search if climate impacts are to be considered in evidence-based decisions. Therefore, the title of an economic evaluation should include terms that enable readers to identify that climate impacts are included, e.g., terms like "cost-effectiveness" combined with "climate footprint". | A climate-extended cost-utility analysis of four different diabetes prevention in individuals with obesity. |
|  | **Abstract** |  |  |  |  |
| **2** | Abstract | Provide a structured summary that highlights context, key methods, results, and alternative analyses. | Yes | Adding climate impacts to health economic evaluations involves at least extending the abstract’s methodology and results section. The method for assessing greenhouse gas (GHG) emissions and converting them to costs or outcomes should be described along with the adjusted cost or outcomes. | Objectives: […] We conduct a climate-extended economic evaluation, comparing usual care four different interventions designed to promote weight loss and diabetes prevention in individuals with obesity. The interventions compared include: A-health promotion focusing on the plant-based diet, B- intensive nutritional counselling for the planetary health diet, C- a CO_2_-intensive formula diet, and D-psychotherapy aimed at behavioural change towards a low-carb diet. […]  Methods: […] We estimated Greenhouse Gas Emissions (GHG) using life-cycle assessment methods. Background data was taken from the ecoinvent database (emissions were converted by factors taken from the literature). We converted the GHG into climate costs, based on the non-traded price of carbon (nTPC) of €189.6 and in DALYS based on ReCiPe […]  Results: […] Compared to the base case, the ICER-Climate Cost extended (ICER-CCE) of intervention B is €10,895/DALY^[1]^ , intervention C is €14,587/DALY and intervention D is €165,872/DALY. The NMB-CCE of Intervention A is €95,758.4 of intervention B is €186,584 of intervention C is €206,208 and intervention D is €197,620.8 […] |
|  | **Introduction** |  |  |  |  |
| **3** | Background and objectives | Give the context for the study, the study question, and its practical relevance for decision-making in policy and practice*.* | Yes | In health economic evaluations, methodological decisions and assumptions have to be made depending on the research question and decision context. This decision context may be influenced by the climate crisis and politically defined GHG reduction targets. The ISO standard for CF requires defining a goal that includes the following: “The intended application, the reasons for carrying out the CFP study, the intended audience, the intended communication” (ISO14067:2018-3.5) see cf.International Organization for Standardization [1] The International Life Cycle Data System (ILCD) handbook distinguishes at least 22 different applications. Examples are a simplified life cycle assessment (LCA) for a quick overview (e.g., in early design stages), policy information (e.g., identifying product groups with the largest environmental impact and also improvement opportunities), or making the supply chain sustainable.[2] The reasons for carrying out the study should include the driving forces and the motivations and should identify the decision context.^8^ The presentation of the results should be tailored to the intended audience, which can be internal or external or have a technical or non-technical background.[2] Intended communication means if whether the results are intended for publication (ISO 14040:2006-4.2.2).[3] Given that the intended application alongside HEE is typically comparison of products and the intended communication is typically scientific publication, at least the reason for assessing CF and the intended audience should be addressed. | Our aim is to enable decision-makers to consider climate impacts by providing scientific evidence and identifying effective health services with low-carbon emissions. This follows the demand for climate-smart health care which was the commitment of the Alliance for Transformative Action on Climate and Health (ATACH[4] and the Declaration Of The Seventh Ministerial Conference On Environment And Health [5, 6].[…]  The objective of the study is to compare four interventions for weight-loss, in terms of monetary-, climate impacts, and health effects. The interventions include: A- health promotion focusing on the plant-based diet, B – intensive nutritional counselling tailored to the planetary health diet, C- a CO_2_-intensive formula diet, and D- psychotherapy aimed at encouraging behavioural change towards a low-carb diet. This evaluation will help in determining the most sustainable and effective approach to combating obesity and preventing diabetes. The results of the comparison between the four alternating interventions are reported via a scientific article and are relevant for governmental politicians and decision-makers within health insurance. |
|  | **Methods** |  |  |  |  |
| **4** | Health economic analysis plan | Indicate whether a health economic analysis plan was developed and where available. | No | This item applies to economic evaluations with and without climate impacts equally. | N.A. |
| **5** | Study population | Describe characteristics of the study population (such as age range, demo­graphics, socioeconomic, or clinical characteristics)*.* | Optional | The study population (cf. “variables such as income, household size occupation, age, household composition, housing area and area of residence”)[7] can influence the results, not only for the measurement of health effects in economic evaluation but also for the measurement of greenhouse gas emissions. | The study population consists of obese adults over the age of 18 years. Climate impacts may be affected by different life patterns within different subgroups. |
| **6** | Setting and location | Provide relevant contextual information that may influence findings*.* | Yes | Considering climate impacts makes additional contextual information relevant. This is because climate impacts accrue both inside and outside the health system. The five phases of a product’s life cycle (raw material extraction, manufacturing, distribution, use, disposal/recycling (ISO 14067:2018-5.2)[1] can take place in different locations (for instance, the production phase in China, the usage phase in Germany). This may involve differences in electricity mix, mobility behavior, or trade relations. Also, climate impacts may differ between rural and urban settings, since, for example, physicians potentially have higher travel costs/fuel consumption in rural areas. Also, different regions within one country may differ regarding waste management (landfill disposal, incineration, recycling). Hence, also the relevant contextual information about the environmental impacts ought to be provided. | Since Germany is the location for the intervention, we also use the German electricity mix. For produced inputs, no specific information about the location of production was available. Therefore, we have used global averages. |
| **7** | Comparators | Describe the interventions or strategies being compared and why chosen*.* | Yes | Describing the objects of investigation is also an important item in CF analyses. Compared to standard information in economic evaluations (description of delivery and content of an intervention), it requires additional information (such as the product system, system boundary, and reference flows). A product system is the totality of connected processes (series of interrelated mechanisms that convert inputs and outputs [ISO 14067:2018-3.1.3.5])[1] and flows (material or energy that enters or leaves the system under study without human treatment [ISO 14067:2018-3.1.3.10])[1]. Not every single process can be included in a life cycle model; therefore, a system boundary based on a set of criteria is needed (ISO 14067:2018-6.3.4.7).[1] This set of criteria should be clearly described (ISO 14040:2006-5.2.3).[8] The reference flow is the starting point of the modeling processes required to obtain the product under investigation. The reference flow is a flow (product or service) that represents the whole product system and is required to fulfill the function of the system expressed by the functional unit (see item 11), i.e., to achieve the target of the intervention itself (ISO 14067:2018-3.1.3.9).[1] “Reference flow” and “description of comparators” (including a description of the resources used) are thus very similar concepts. | Intervention A is health promotion focusing on the planetary health diet, intervention B is intensive nutritional counselling tailored to the planetary health diet, Intervention C is a CO_2_-intensive formula diet, and intervention D is psychotherapy aimed at behavioural change towards a low-carb diet. Regarding the system boundaries to calculate the climate impacts, we included all life-cycle phases.  We exclude for the research object of formula diet all processes connected with the production of machinery (e.g. machinery in the production, ships for transports) as to the longevity, the emissions per produced or delivered products are assumed negligible. Furthermore, we excluded all processes connected with delivery from the store to the customer as the uncertainties would be too high. The product system is visualized with openLCA (…). |
| **8** | Perspective | State the perspective(s) adopted by the study and why chosen*.* | Yes | Generally, this item applies to economic evaluation with and without climate impacts equally because climate impacts can be added to each adopted perspective. If environmental impacts were included that incur local external costs (like, e.g., sooth particles), then distinguishing between the perspectives of internal and external costs and reporting both is advisable. | (For this study we chose the societal perspective and add climate impacts to expenses of all payers.) |
| **9** | Time Horizon | State the time horizon and why appropriate. | Yes | In standard economic evaluation, the time horizon is defined as the period for which monetary costs and health effects are calculated. Given that climate impacts generally occur after GHG emissions, including climate impacts adds further dimensions to the time horizon. This is also because the duration of GHG in the atmosphere varies and the decision about the considered time is connected to normative values. Typically, time horizons distinguish between radiation potential over the next 20, 100, or 1000 years. This choice of time horizon is made in conjunction with the choice of characterization factors that compare the radiation potential of different GHGs (see Item 15). Given that the standard GWP time horizon of 100 years is similar to the standard life time horizon of HEE, at least, reasons for deviating from this value or the time horizon chosen for health effects, outcomes, and related costs should be explained. | For this study, monetary costs, disease costs and health effects are calculated over a lifetime horizon, because we expect long-term health gains by a lower risk for diabetes. The choice of time horizon for GHG are made in conjunction with the choice of characterization factor, which is a global warming potential of 100 years. |
| **10** | Discount rate | Report the discount rate(s) and the reason chosen. | Yes | Discounting costs and consequences in economic evaluations is an issue of intense debate[9]. Likewise, in the LCA literature, static and dynamic models are distinguished and dynamic models include discount rates for future impacts (see cf.Pehnt [10]) Particularly, if impacts are expressed as costs like the social cost of, e.g., carbon[11] (see also: Item 15), discounting appears highly relevant. However, discounting is still a matter of debate as it is frequently seen as conflicting with the principle of intergenerational equity[12]. Most frequently, LCAs as well as most LCA databases are based on static models. | The discount rate for the costs of the intervention is chosen to be 3% according to the recommendations of the Independent Institute for Quality and Efficiency in Healthcare (IQWIG) [13]. The discount rate for the physical emissions is chosen as 0% to account for ethical concerns against disadvantaging future generations [14]. We therefore chose a static LCA model. However, for the unification of costs, we chose social cost of carbon, social cost of methane and social cost of nitrous oxide (see 15) we also chose a discount rate of 3%. |
| **11** | Selection of outcomes | Describe what outcomes were used as the measure(s) of benefit(s) and harm(s). | No | Selection of outcomes is not only an important item that determines the type of economic evaluation (e.g., if an economic evaluation selected QALYs as the outcome to conduct a cost–utility analysis[15]), it is also linked to an important methodological choice of CF calculation: the definition of the functional unit. The functional unit makes consistent comparisons between interventions possible. The functional unit (e.g., measurement of heart rate data of a wearable device over three months) is defined as the quantified performance of a product system (e.g., collection of processes and flows performing the function of a wearable device [see item 7]) for use as a reference unit (see: ISO 14067:2018-3.1.3.7).[1] The specified outcomes of the health economic evaluation form the basis for the calculation of the climate impacts. In addition to the calculation of monetary units per outcome, this also enables the calculation of climate impacts per outcome. Therefore, we use the terms “definition of functional unit” and “selection of health outcomes” interchangeably. As health outcomes are to be reported in every full HEE, there is thus nothing to extend for including climate impacts.  If the health economic evaluation assesses cost per DALY, subtracting health decrements from climate impacts from an intervention’s health benefits appears straightforward. In other cases, further detail is necessary to demonstrate the validity of aggregating health benefits and decrements. | The health outcome of the intervention is measured in weight loss in kg. Based on this, we modelled the weight loss in kg to DALYs. To calculate climate impacts, we define the functional unit as: “one DALY gained” and also incorporate climate impacts as DALYs. |
| ***11a*** |  |  | *Yes* | If the health economic evaluation assesses cost per DALY, subtracting health decrements from climate impacts from an intervention’s health benefits appears straightforward. In other cases, further detail is necessary to demonstrate the validity of aggregating health benefits and decrements. | We incorporate climate impacts as DALYs. |
| **12** | Measurement of outcomes | Describe how outcomes used to capture benefit(s) and harm(s) were measured. | No | This item applies equally to economic evaluation with and without climate impacts. | N.A |
| **12a** | Measurement of climate outcomes |  | Yes | If climate impacts are incorporated as costs, there are no climate-related modifications in relation to this item. If they are incorporated as health outcomes, life cycle impact assessment models of ReCiPE,[16] UseTox,[17] ILCD,[2] Impact 2002+,[18] and EcoIndicator99[19] include the model-based conversion of climate impacts into DALYs that can be incorporated into assessments of cost per DALY. Additionally, damage factors from the literature can be used to convert greenhouse gas emissions into health impacts in terms of DALYs.[20] | DALYs were calculated by using the latest IPCC characterisation factors, and then converting those to DALYs with Tang et al [20] |
| **13** | Valuation of outcomes | Describe the population and methods used to measure and value outcomes. | Yes | This item applies to equally economic evaluations if climate impacts are incorporated as costs. If outcomes are incorporated as benefits, it is most likely that both the intervention effects and impacts of climate change are reported as DALYs using disability weights of the Global Burden of Disease Reports. Different from the values of health states using QALYs, these DALY weights do not differ across jurisdictions or cultural contexts.[22] | We used the disability weights for the Global Burden of Disease Reports [23]. |
| **14** | Measurement and valuation of resources and costs | Describe how costs were valued. | Yes | In the former "Drummond-Checklist", costing in economic evaluation was based on identifying and measuring relevant resource use in physical units. These measured Physical units could also provide the most precise basis for measuring climate impacts (item 14a), and valuing climate costs (item 14b). Examples include the download of a health promotion app to promote physical activity or five one-hour online calls by a nutritionist. Frequently, reimbursement rates are used as a proxy of costs from a healthcare system perspective. This omits the step of measuring resources in physical units and lacks precision if reimbursement rates cover different types of technology or human activities that involve different climate costs.  Sometimes, resources that serve many different departments, programs, or processes; for example, general hospital administration or power consumption (so-called overhead costs).[24] One opportunity to avoid overhead costs is to allocate the overhead costs of one department to another (called allocation basis).[24] Allocation in climate footprinting follows a specified order of methods: First, avoiding allocation by dividing the process into sub processes or system expansion; second, allocation based on the underlying physical relationships; and, third, allocation based on relationships other than physical; for example, “input and output data might be allocated between co-products in proportion to the economic value of the products” (ISO 14067:2018-6.4.6.2).[1] After measuring resources in physical units the amounts could be multiplied by adequate unit costs or prices. The unit costs or prices depend on the adopted perspective (see Item 8).[24] | The intervention includes health promotion that focuses on adopting the plant-based diet (intervention A), intensive nutritional counseling specifically tailored to support and educate on the planetary health diet (intervention B), a CO_2_-intensive formula diet (intervention C), and psychotherapy aimed at facilitating behavioral change towards adopting a low-carb diet (intervention D). Resource use in physical units is estimated using expert interviews, complemented by literature search.  (…) Intervention C involves a formula diet available for purchase at pharmacies-or drugstore- (websites). The package consists of the package, the packaging of the formula diet, the powdered formula diet and instruction for use. We assume that patients will order it online. In order, to calculate the carbon footprint of the transport to the patient´s home, the weight of the package, the booklet, and the packaging of the formula diet product was measured.  Intervention D comprises three consultations with the psychologists, which only include electricity and paper consumption. Patients were assumed to travel to the consultation as part of their daily travel to work. To avoid counting the whole trip to the intervention, only the portion outside their normal travel (i.e. to the psychologist) was accounted for (ISO 14067:2018-6.4.6.2) [1]. (…)  For the monetary valuation of the formula diet we used the prices of the producer. Based on literature search we assume costs for the intervention C: formula diet (€22), shipping (€4.99).  For intervention D, according to the German EBM catalogue (forms the basis for the billing of services provided by accredited physicians and psychotherapists)[25] the cost of one hour of psychotherapy is €112,30 (EBM 35405). Travelling costs per kilometer are assumed in the amount of €1 (Public transport), taxi €5 (taxi) and car €0.33 (fuel price per km (€1.60) and gasoline consumption of 7 liters per 100 km). (…) |
| **14a** | *Measurement of climate impacts* |  | *Yes* | The IPCC recommends including the greenhouse gases carbon dioxide (CO2), methane (CH4), nitrous oxide (N2O), hydrofluorocarbons (HCFCs), perfluorocarbons (PFCs), and sulfur hexafluoride (SF6).[26]  There are different options for assessing the amount of GHGs emitted using life cycle assessment. First, based on the measurement of resources in physical units (14), process-based CF calculate emissions based on physical resources, based on an inventory of energy and materials required throughout the life cycle of a product or process, which is standardized by ISO-Standard 14040/14044/14067. Emission data can be taken from LCA databases (which is preferable), from published LCAs, or own measurements.  Second, if no such information is available, GHG emissions can be calculated by multiplying units’ costs (14) and cost-based emission factors. These emission factors are based on input-output-life cycle assessments (I-O-LCAs). IO-LCAs analyze the economic transactions between industries and sectors. IO-LCA is based on the idea that every product or service requires inputs from other industries, which in turn require inputs from other industries, and so on. By tracking these inter-industry flows of materials and energy, IO-LCA can estimate the climate footprint of a product or service across the entire supply chain. However, as emission factors only exist for industries, aggregation errors can lead to higher uncertainty.[27] | Emission quantities for the formula diet were taken from the manufacturer´s information (26 kg CO2 . The psychotherapeutic consultation generates due to the use of electricity and paper 1kg of CO_2_. The trip to the psychotherapists on feet or by bicycle has no CO_2_ emissions. For the public transport; taxi or car, the researcher chose the emission factors by the database exiobase and ecoinvent [28, 29]: The tram emits per 0.478 kg CO_2_ and 0.00122 kg CH_4_ per € 1.00. The car and taxi emit per 0.237 kg CO_2_ per €1.00, 0.000005893 kg CH4 and 0.0000000008640 (Car Euro 3) per km referred to ecoinvent (…)We include all six greenhouse gases reported by the intergovernmental panel on Climate Change (IPPC) [30]. |
| **14b** | *Valuation of climate costs* |  | *Yes* | To add climate costs to the intervention costs, there are two opportunities to assign a monetary value to GHG emissions. First, based on the measured GHG emission in item 14a and converted to an overall measure GHG in CO_2_eq, it can be multiplied with a related price of carbon. A possible price for this would be the social cost of carbon, which measures and monetizes the total net damages caused by an additional metric ton of CO_2_eq emissions due to climate change. Given that the social cost of carbon excludes emissions that are already subject to taxation or emission trading schemes, this value needs to be corrected to a non-traded price of carbon (nTPC).  Second, in cases where greenhouse gases are not aggregated to CO_2_eq, a price can be assigned to each individual greenhouse gas. This approach can be justified by the fact that greenhouse gases other than CO_2_ are not currently subject to taxation or trading. Hereby, the chosen social price of carbon will be multiplied by the global warming potential (GWP). GWP is an index, that measures the radiation potential (i.e. the GHG´s impact on global warming) in the atmosphere compared with the radiation potential of CO_2_ (ISO 14067:2018-3.1.2.4).[1] For CH_4_ (methane), this corresponds to 28 times the rate of the CO_2_eq cost rate, for N_2_O (nitrous oxide) 265 time the rate.[31] Based on this calculation, the measured GHG emission (item 14a) can be multiplied with the social price of the corresponding GHG. In this case, the conversion (item 15) would be redundant. Finally, it can be aggregated to obtain the sum of all climate costs of the intervention. | We have opted to include climate impacts as climate cost in accordance with the ISO 14007 and 14008 [32, 33] and the recommendation of Fraas, Graham [34]. Therefore, we orientate for the nTPC for a ton of CO_2_eq on the social cost of carbon recommendation by the German Environment Agency [31] for 2022 and the reduction recommendation of Shukla, Skea [35] a value of €189,6 . We converted CO_2_, HCFs, PFCs and SF6 into nTPC, whereas we used for CH_4_ and N_2_O the social cost of it referring Matthey and Bünger [31]. |
| **15** | Currency, price date, and conversion | Report the dates of the estimated resource quantities and unit costs, plus the currency and year of conversion. | Yes | Similar to different currencies that can be used for monetary valuation and for converting different values, emission data from the climate valuation are available for various GHGs. In cases where calculating each individual GHG is impractical, emissions can be transformed into a common unit, namely CO_2_ equivalents (CO_2_eq). The characterization factor of each GHG specifies the radiation potential compared with the radiation potential of CO_2_. It is expressed in CO_2_eq, which is the global warming potential (GWP) of a GHG multiplied by the mass of the GHG in units of the potential of CO_2_ (ISO 14067:2018-3.1.2.2).[1] Characterization factors are usually taken from established databases such as the emission factor database (EFDB) of the IPCC[36] or from established characterization models such as the ReCiPe model.[37]. | DALYs and climate impacts were calculated by using ReCiPe [16]. |
| **16** | Rationale and description of model | If modelling is used, describe in detail and why used. Report if the model is publicly available and where it can be accessed. | Optional | This item applies to economic evaluations with and without climate impacts equally. Generally, LCA can be seen as a modelling method on its own. It could even be the only modelling applied in a study - for example, if a trial-based economic evaluation includes climate impacts that stem from LCA models of certain resources used within the study. However, this topic is accounted for in item #14b to retain a focus on health economic evaluation methods. Therefore, only any further modelling to estimate CF should be reported under this item. | N.A |
| **17** | Analytics and assumptions | Describe any methods for analyzing or statistically transforming data, any extrapolation methods, and approaches for validating any model used. | Optional | Climate-extended economic evaluations involve analytics and assumptions. However, the most important ones are covered elsewhere. In particular, these include the system boundary (see item 7), characterization model (see item 15), representativeness of the data (see item 20), and comparability of production systems in terms of functional units (see item 11). Furthermore, extrapolation of data related to CF may be needed as for other parameters. Therefore, this item applies to economic evaluations with and without climate impacts equally even if it might provide a valuable reminder to add further specific analytics and assumption that are not yet covered. | (We extrapolated the route to the nutritional counselling and psychotherapy on the basis of the German modal split and the duration of the respective intervention). |
| **18** | Characterizing heterogeneity | Describe any methods used for estimating how the results of the study vary for subgroups. | Optional | Heterogeneity in CF can be highlighted by reporting the CF variations of different consumption/usage patterns of the individual study population group. However, unless specific heterogeneity regarding CF is expected, this item applies to economic evaluations with and without climate impacts equally. | (The traffic behavior could be reported by subgroups. On this basis, the CF per subgroup could be calculated and heterogeneity characterized.) |
| **19** | Characterizing distributional effects | Describe how impacts are distributed across different individuals or adjustments made to reflect priority populations. | Optional | Equality and equity are well-known issues in the evaluation of health services. If climate costs are integrated, this can also be considered when calculating them, because the consequences of climate change are global and occur regardless of where greenhouse gases are emitted. Nevertheless, there are global differences in prosperity that lead to different perceptions of climate damage.[38] These differences in wealth can be taken into account by using equity weighting. For example, the monetary values of the local damage caused can be weighted by the average income of the country. However, the use of equity weighting in the calculation of climate costs is not common. It would not be necessary to report if the injured parties were actually compensated immediately by those who caused the damage.[31] | (The intervention is limited to Germany. Therefore, we assume that climate damage affects the entire population equally.) |
| **20** | Characterizing uncertainty | Describe methods to characterize any sources of uncertainty in the analysis. | Yes | Accounting for climate impacts introduces new sources of uncertainty into the analysis that ought to be addressed. The ISO norm requires characterizing the quality of data in terms of aspects like time-related coverage (i.e. that age of the data and length of data collection is appropriate), geographical and technology coverage (i.e. that the data are applicable to the geographical areas of the unit processes or the technologies), or completeness (i.e. percentage of the measured flow which is covered by the data) (ISO 14067:2018-6.3.5).[1] The ISO-Standard 14067 does not specify how to verify the uncertainty of the data, only that researchers calculating a climate footprint should have a system in place to do so.  A tool recommended for LCAs is the Pedigree matrix. It involves rating the data quality from very good to very poor data quality for the five criteria of Reliability, Completeness, Temporal correlation, Geographical correlation, further technological correlation.[39] Using uncertainty scaling factors for each data quality ranking, it can be used to assign probability distributions to the data. Most LCA software supports the use of pedigree matrixes. However, the total uncertainty of the process can only be calculated, if the uncertainty of the inputs is also known. This is more often the case if LCA databases are used than published LCAs. Alternatively, uncertainty in data can be described in a qualitative manner.[2] | Sources of uncertainty for the CF are presented in the Pedigree matrix format and analyzed the data in accordance with [39]: Uncertainty analysis was carried out by using the results of the Pedigree Matrix using a 95% confidence interval of 1500 Monte Carlo simulation runs using OpenLCA software. Furthermore, a tornado diagram was also created for the sensitivity analysis. The processes were reviewed and documented by two types of third-party reviewers. |
| **21** | Approach to engagement with patients and others affected by the study | Describe any approaches to engage patients or service recipients, the general public, communities, or stakeholders (such as clinicians or payers) in the design of the study. | No | The ISO-Standard 14044 (ISO 14044:2018-4.2.3.7)[3] recommends protecting stakeholders when comparing two product systems. In addition, the ILCD-Handbook also recommends taking stakeholders into account when documenting study results to avoid misinterpretation by non-technical audiences.[2] Nevertheless, this item applies to economic evaluations with and without climate impacts equally. | N.A |
|  | **Results** | . |  |  | N.A. |
| **22** | Study parameters | Report all analytic inputs (such as values, ranges, references) including uncertainty or distributional assumptions. | Yes | In the LCA literature, the report of all analytic inputs typically takes the form of a life cycle inventory model (LCI). LCI involves creating an inventory of inputs and outputs for a product system and is usually performed in specific LCA software (e.g., openLCA) or, for smaller inventories, as spreadsheets. The inventory can be based on literature analysis or process simulation. | Table 3 lists (1) resource item, (2) amount in physical units, (3) source of amount, (4) costs in EUR, (5) assumed distribution, (6), parameters of cost distribution, (7) source of costs, (8) CO_2_eq per unit, (9) relevant units (costs or physical unit), (10) distribution of emissions, and (11) source of distribution.  The results of the CF are following: (…) The psychological consultation is because of three further check-up multiplied to total 19.6 kg CO_2_. The route to the psychologists are conducted cost-based and multiplied by six (roundtrip a 3 times) for the tram to 8.604 kg CO_2_ and 0.00732kg CH_4_. The car would emit 0.4692 kg CO_2_ and the taxi would have an output of 17.2062 kg CO_2_.Furthermore, a Euro 3 car (whether taxi or private) would emit for the whole trip 0.000106074 CH_4_ and 1.5552e-8 N_2_O. (…)  After the conversion by the characterization factors and the multiplication with nTPC of 200€ the intervention C would emit 20 t CO_2_eq and thus cost €4,000. Intervention D 2t CO_2_eq and cost €400 (…) |
| **23** | Summary of main results | Report the mean values for the main categories of costs, and outcomes of interest and summarize them in the most appropriate overall measure. | Yes | As requested by CHEERS 2022, mean values and outcomes should be summarized in “the most appropriate overall measure.” Various methods can be used to include climate impacts as costs or outcomes in overall measures. For example, if climate impacts are integrated as an outcome on the effect side (see item 11a), a climate-effectiveness-extended ICER (ICER-CEE) (e.g., €/QALY) can be reported. Without integration, the incremental carbon footprint effectiveness ratio (ICFER) ratio can be reported in terms of tons of CO_2_eq per health outcome. If climate impacts are transformed into climate costs, these can be added to the cost side of the ICER and reported as climate (-cost-) extended ICER (ICER-CCE). Furthermore, net monetary benefits (NMBs) based on incremental costs, effects, and a threshold value^19^ can be climate-extended (NMB-CCE) by adding climate costs to the conventional costs or climate-effectiveness-extended by adding climate impacts converted into outcomes (NMB-CEE). Likewise, net benefits from cost–benefit analyses can be climate (effectiveness)-extended (NB-CE and NB-CEE) in the same way. For efficient climate change mitigation, it is useful to analyze which component of the studied intervention was the largest CO2eq emitter (so-called hotspot analyses, cf.Bjørn, Owsianiak, Laurent, et al. 34) Therefore, the climate impacts can also be reported per component, if the emission of GHGs was particularly high, as described in item 18. | The ICER of intervention C is €10,000/DALY, whereas the ICER of intervention D is €200,000/DALY. The ICFER of intervention D is 0.65t CO_2_eq/DALY. Adding climate costs, the ICER-CCE for intervention B is €10,895/DALY, intervention C is €14,587/DALY and intervention D is €165,872/DALY. The NMB of intervention A is €95,000, of intervention B €17,000, of intervention C is €210,000 and of intervention D is €198,000. The NMB-CCE of intervention A is €95,758, of intervention B €186,584, of intervention C is €206,208 and of intervention D is €197,620. When integrating the climate impacts on the outcome side the ICER-CEE of intervention C is €10,062/DALY and for intervention D is €171,374/DALY. The NMB-CEE is €95,296 for intervention A, €181,970 for intervention B, €211,485 for intervention C and €198,148 for intervention D. The hotspot analysis showed that the use of a car to the counselling and psychotherapy contributes most to CF. |
| **24** | Effect of uncertainty | Describe how uncertainty about analytic judgments, inputs, or projections affects findings. Report the effect of choice of discount rate, time horizon, if applicable. | Yes | Methodological requirements for characterizing uncertainty are described in item 20. The effect of uncertainty also regarding aspects relevant for CF ought to be addressed as well. | N.A. |
| **25** | Effect of engagement with patients and others affected by the study | Report on any difference the patient/service recipient, general public, community, or stakeholder involvement made to the approach or findings of the study. | No | This item applies to economic evaluations with and without climate impacts equally. | N.A |
|  | **Discussion** |  |  |  |  |
| **26** | Study findings, limitations, generalizability, and current knowledge | Report key findings, limitations, ethical or equity considerations not captured, and how these could affect patients, policy, or practice. | Yes | As with health economic evaluations, limitations of the climate footprint study should be reported. For climate footprint, limitations are defined by the ISO Norm 14067 (ISO 14067:2018-Annex A).[1] The two most important limitations are the focus on climate change as the single impact category, and limitations related to methodology, such as choice of system boundary and functional unit; data quality and data sources; allocation procedure; and assumptions about transport, user behavior, or end-of-life scenarios (ISO 14067:2018-Annex A-A1-A3).[1] | The estimation of CF had to be based on limited data because especially the scope 3 data of the formula diet were not traceable. Future work is needed to fill up this gap. |
|  | **Other relevant information** |  |  |  |  |
| **27** | Source of funding | Describe how the study was funded and any role of the funder in the identification, design, conduct, and reporting of the analysis. | No | This item applies to economic evaluations with and without climate impacts equally. | N.A |
| **28** | Conflicts of interest | Report authors conflicts of interest according to journal or International Committee of Medical Journal Editors requirements. | No | This item applies to economic evaluations with and without climate impacts equally. | N.A |

1.	International Organization for Standardization, *Greenhouse gases - Carbon footprint of products -Requirements and guidelines for quantification - DIN EN ISO 2018 14067*. 2019.

2.	European Commission *International Reference Life Cycle Data System (ILCD) Handbook- General guide for Life Cycle Assessment* 2010, Joint Research Centre - Institute for Environment and Sustainability: Luxembourg.

3.	International Organization for Standardization, *Environmental management-Life cycle assessment -requirements and guidelines - DIN EN ISO 14044* 2018.

4.	World Health Organization, *Final Terms Of Reference Of The Alliance For Transformative Action on Climate And Health (ATACH)*. 2022.

5.	World Health Organization, H.C.W.H., UN *COP 26 Health Programme - Country Commitments to build climate resilient and sustainable health systems* 2021.

6.	World Health Organization, *Declaration Of The Seventh Ministerial Conference On Environment And Health*. 2023: Budapest, Hungary.

7.	Mahía, R. and R. de Arce, *Quantifying the excess carbon footprint and its main determinants of Spanish households.* Energy & Environment. **0**(0): p. 0958305X221140582.

8.	International Organization for Standardization, *Environmental management - Life cycle assessment - Principles and framework -DIN EN ISO 14040* 2006.

9.	Attema, A.E., W.B. Brouwer, and K. Claxton, *Discounting in economic evaluations.* Pharmacoeconomics, 2018. **36**: p. 745-758.

10.	Pehnt, M., *Dynamic life cycle assessment (LCA) of renewable energy technologies.* Renewable energy, 2006. **31**(1): p. 55-71.

11.	Nordhaus, W., *Estimates of the social cost of carbon: concepts and results from the DICE-2013R model and alternative approaches.* Journal of the Association of Environmental and Resource Economists, 2014. **1**(1/2): p. 273-312.

12.	Lueddeckens, S., P. Saling, and E. Guenther, *Discounting and life cycle assessment: a distorting measure in assessments, a reasonable instrument for decisions.* International Journal of Environmental Science and Technology, 2021: p. 1-12.

13.	IQWIG, *Allgemeine Methoden* 2022.

14.	Hellweg, S., T.B. Hofstetter, and K. Hungerbuhler, *Discounting and the environment should current impacts be weighted differently than impacts harming future generations?* The International Journal of Life Cycle Assessment, 2003. **8**: p. 8-18.

15.	Drummond, M.F., et al., *Methods for the economic evaluation of health care programmes*. 2015: Oxford University Press.

16.	Huijbregts, M.A., et al., *ReCiPe 2016: a harmonized life cycle impact assessment method at midpoint and endpoint level report I: characterization.* 2016.

17.	Rosenbaum, R.K., et al., *USEtox—the UNEP-SETAC toxicity model: recommended characterisation factors for human toxicity and freshwater ecotoxicity in life cycle impact assessment.* The International Journal of Life Cycle Assessment, 2008. **13**(7): p. 532-546.

18.	Jolliet, O., et al., *IMPACT 2002+: A new life cycle impact assessment methodology.* International Journal of Life Cycle Assessment, 2003. **8**(6): p. 324-330.

19.	Goedkoop, M.J., *The Eco-indicator 99 a damage oriented method for life cycle impact assessment methodology report.* Pre Concultants, 1999.

20.	Tang, L., et al., *Development of human health damage factors related to CO 2 emissions by considering future socioeconomic scenarios.* The International Journal of Life Cycle Assessment, 2018. **23**: p. 2288-2299.

21.	iPoint-systems gmbh. *Life Cycle Impact Assessment- the most widely used LCIA Methods* 2018 13.09.2024]; Available from: <https://go.ipoint-systems.com/blog/lcia-indicator>.

22.	Salomon, J.A., et al., *Common values in assessing health outcomes from disease and injury: disability weights measurement study for the Global Burden of Disease Study 2010.* The Lancet, 2012. **380**(9859): p. 2129-2143.

23.	Institute for Health Metrics and Evaluation (IHME), *Global Burden of Disease 2021: Findings form the GBD 2021 Study* 2024.

24.	Drummond, M.F., et al., *Methods for the economic evaluation of health care programmes*. 2015, Oxford University Press: Oxford.

25.	Bundesministerium für Gesundheit. *Einheitlicher Bewertungsmaßstab-EBM*. Begriffe von A-Z 2023; Available from: <https://www.bundesgesundheitsministerium.de/service/begriffe-von-a-z/e/einheitlicher-bewertungsmassstab-ebm.html#:~:text=Der%20einheitliche%20Bewertungsma%C3%9Fstab%20(EBM)%20bildet,und%20des%20GKV%20-Spitzenverbandes%20zusammensetzt>. .

26.	UNFCCC, *Kyoto Protocol to th United Nations Framework Convention on Climate Change* 1997.

27.	Smith, C.L., Y. Zurynski, and J. Braithwaite, *We can’t mitigate what we don’t monitor: using informatics to measure and improve healthcare systems’ climate impact and environmental footprint.* Journal of the American Medical Informatics Association, 2022. **29**(12): p. 2168-2173.

28.	Stadler, K., Wood, R., Bulavskaya, T., Södersten, C.-J., Simas, M., Schmidt, S., Usubiaga, A., Acosta-Fernández, J., Kuenen, J., Bruckner, M., Giljum, S., Lutter, S., Merciai, S., Schmidt, J. H., Theurl, M. C., Plutzar, C., Kastner, T., Eisenmenger, N., Erb, K.-H., . . . Tukker, A.,, *EXIOBASE 3: Developing a Time Series of Detailed Environmentally Extended Multi-Regional Input-Output Tables.* Journal of Industrial Ecology, 2018. **22(3)**: p. 502-515.

29.	Wernet, G., et al., *The ecoinvent database version 3 (part I): overview and methodology.* The International Journal of Life Cycle Assessment, 2016. **21**: p. 1218-1230.

30.	IPCC, et al., *Climate Change 2013: The Physical Science Basis. Contribution of Working Group I to the Fifth Assessment Report of the Intergovernmental Panel on Climate Change* in *Cambridge University Press*. 2013. p. 1535.

31.	Matthey, A. and B. Bünger, *Methodenkonvention 3.1 zur Ermittlung von Umweltkosten Kostensätze Stand 12/2020*. 2020, Umweltbundesamt: Dessau-Roßlau.

32.	International Organization for Standardization, *Environmental management - Guidelines for determining environmental costs and benefits* in *ISO 14007:2019*. 2019.

33.	International Organization for Standardization, *ISO 14008 Monetary valuation of environmental impacts and related environmental aspects -DIN EN ISO 14008:2019(E)*. 2019.

34.	Fraas, A.G., et al., *Seven recommendations for pricing greenhouse gas emissions.* Journal of Benefit-Cost Analysis, 2023. **14**(2): p. 191-204.

35.	Shukla, P.R., et al., *Climate Change 2022 Mitigation of Climate Change. Working Group III Contribution to the Sixth Assessment Report of the Intergovernmental Panel on Climate Change. Summary for Policymakers*. 2022, [IPCC]: [Geneva].

36.	Intergovernmental Panel on Climate Change, *emission factor database (EFDB)*. 2023.

37.	Goedkoop, M., et al., *ReCiPe 2008.* A life cycle impact assessment method which comprises harmonised category indicators at the midpoint and the endpoint level, 2009. **1**: p. 1-126.

38.	IPCC, et al., *Climate Change 2022 Mitigation of Climate Change - Working Group III Contribution to the Sixth Assessment Report of the Intergovernmental Panel on Climate Change - Summary for Policymakers.* 2022.

39.	Weidema, B.P. and M.S. Wesnæs, *Data quality management for life cycle inventories—an example of using data quality indicators.* Journal of cleaner production, 1996. **4**(3-4): p. 167-174.

1. All numbers are fictitious and are provided solely for illustrative purposes.
