# Supplementary material for: Points to consider for incorporating climate impacts into health economic evaluation
Source: Eur J Health Econ. 2026 Jan 7;27(6):1261–76. doi: 10.1007/s10198-025-01859-3 (PMC13451359; doi:10.1007/s10198-025-01859-3)
Supplement: Supplementary file 3 [file 10198_2025_1859_MOESM3_ESM.docx]

Table 5 Comparison of ICER, NMB, ICFER, ICER-CCE, NMB-CCE, ICER-CEE, NMB-CEE with the values of EPH [118]

| **ΔDALYs** | **ΔCosts** | **ICER** | **NMB** | **ΔtCO2eq** | **ICFER** | **Costs-CCE** | **ICER-CCE** | **NMB-CCE** | **DALYS-CO2** | **DALY CEE** | **ICER-CEE** | **NMB-CEE** |
| --- | --- | --- | --- | --- | --- | --- | --- | --- | --- | --- | --- | --- |
| 0 | 0 | N.A. | N.A | 0 | N.A. | 0 | N.A | N.A | 0 | N.A | N.A | N.A |
| 1.5 | 25,000 | extendedly dominated | 95,000 | -4 | dominant | 24,242 |  | 95,758 | 0.003712 | 1.504 | extendedly dominated | 95,297 |
| 2.7 | 37,000 | dominated | 179,000 | -40 | dominant | 29,416 | 10,895 | 186,584 | 0.03712 | 2.737 | dominated | 181,970 |
| 3 | 30,000 | 10,000 | 210,000 | 20 | dominated | 33,792 | 14,587 | 206,208 | 0.01856 | 2.981 | 10,062 | 208,515 |
| 3.1 | 50,000 | 200,000 | 198,000 | 2 | 0.65 | 50,379 | 165,872 | 197,621 | 0.001856 | 3.098 | 171,374 | 197,852 |
